# Efficacy of azole therapy for tegumentary leishmaniasis: A systematic review and meta-analysis

**DOI:** 10.1371/journal.pone.0186117

**Published:** 2017-10-09

**Authors:** Endi Lanza Galvão, Ana Rabello, Gláucia Fernandes Cota

**Affiliations:** Pesquisa Clínica e Políticas Públicas em Doenças Infecto-Parasitárias–Centro de Pesquisas René Rachou—Fundação Oswaldo Cruz, Fiocruz, Belo Horizonte, Minas Gerais, Brazil; Academic Medical Centre, NETHERLANDS

## Abstract

**Background:**

Several controlled and uncontrolled studies addressing azole antifungal drugs for cutaneous and mucosal leishmaniasis have been published with inconclusive results. We conducted a systematic literature review of studies evaluating the efficacy and toxicity associated with azole therapy for tegumentary leishmaniasis.

**Methodology:**

PRISMA guidelines for systematic reviews and the Cochrane manual were followed, and the review methodology was registered (PROSPERO; CRD42016048668). Sources included the EMBASE, Web of Science, MEDLINE, LILACS, and IBECS databases along with a manual search of references from evaluated studies. Additional resources such as *Google Scholar* and clinicaltrials.gov were also searched. We included all studies reporting cure rate after cutaneous or mucosal leishmaniasis treatment with systemic azole drugs, regardless of their design. R software was used to estimate global rates of success and adverse events with each drug. The main outcome of interest was clinical cure, defined as complete re-epithelialization of all lesions.

**Results:**

A total of 37 studies involving 1259 patients that reported outcomes after fluconazole (9), ketoconazole (14) and itraconazole (15) treatments were included. Only 14 (38%) were randomized controlled trials (RCT). The pooled azole final efficacy rate was 64% (CI95%: 57–70%) for all studies and 60% (CI95%: 50–70%) (p = 0.41) if only RCTs studies were considered. Twenty-four studies were conducted in the Old World and 13 studies in the Americas. The final efficacy rate according to New and Old World were 62% (CI95%: 43–77%) and 66% (CI95%: 58–73%), respectively. The final efficacy rate of azoles according to species were 89% (CI95%: 50–98%) for *L*. *mexicana*; 88% for *L*. *infantum* (CI95%: 27–99%); 80% for *L*. *donovani;* 53% (CI95%: 29–76%) for *L*. *major*; 49% for *L*. *braziliensis* (CI95%: 21–78%); and 15% (CI95%: 1–84%) for *L*. *tropica*. The cure rates were similar among the fluconazole, ketoconazole and itraconazole group arms (p = 0.89), specifically 61% (CI95%: 48–72%), 64% (CI95%: 44–80%) 65% (CI95%: 56–72%), respectively. Adverse events during fluconazole, itraconazole and ketoconazole therapy were reported in 7% (CI95%: 3–14%), 12% (CI95% 8–19%) and 13% (CI95%: 6–29%) of treated patients, respectively, without difference among them (p = 0.35). This systematic review included studies with small samples and both non-comparative and non-randomized studies and the main limitation was the low quality of the available studies.

**Conclusions:**

Available evidence suggests that fluconazole, ketoconazole and itraconazole have similar and modest efficacy rates for tegumentary leishmaniasis treatment. There is insufficient evidence to support the exclusive use of azole therapy as a single agent for leishmaniasis treatment.

## Introduction

Tegumentary leishmaniasis (TL), comprising cutaneous (CL) and mucosal leishmaniasis (ML), is a parasitic infection caused by protozoa that annually affects 0.7 to 1.2 million people worldwide [1]. Although TL is a non-fatal disease, it is characterized by a broad clinical spectrum involving single or multiple localized skin lesions, severe diffuse and mucosal lesions. TL leads to considerable morbidities and is associated with physical deformities and psychological effects [2–4]. The control of leishmaniasis remains a serious problem, and the few, and often toxic, therapeutic options available are the primary challenge for the disease approach.

There are few drugs available for TL treatment, and some systematic reviews [5–9] have tried to achieve a consensus about an optimal drug treatment for patients using different interventions. Unfortunately, no ideal or universally applicable therapy for leishmaniasis has been identified. The evidence to support the efficacy of different treatments for CL in the Old World is still very limited. In this context, systemic pentavalent antimony (SbV) has remained the first-line treatment for leishmaniasis for decades, achieving a cure rate of 76.5% for American CL [6].

Although SbV is the gold standard therapy, pentavalent antimony cannot be considered a satisfactory option because it requires a daily dosage of injections for 20 to 30 days, it cannot be used in pregnant women, and it has led to severe side effects such as cardiotoxocity and renal failure in several patients [10,11]. Thus, better treatment alternatives are urgently needed.

There is interest in orally administrable antileishmanial agents, and azole therapy comprising fluconazole, ketoconazole and itraconazole may be an alternative that fulfills this requirement. These agents have been shown to be highly efficacious against *Leishmania* spp. in *in vitro* tests [12–14]. Some *in vivo* studies also reported an effective response to this medication group [15–17]. However, the efficacy rate of azole therapy, to the best of our knowledge, has never been compiled.

The aim of this study was to systematically assess, through an evidence-based approach, the efficacy and safety of azole therapy for TL.

## Objectives

Our main objective was to assess the efficacy of azole therapy for TL, following the PICO question: **P**opulation: subjects with TL; **I**ntervention: use of any systemic azole drug; **C**omparison, if applicable: any other therapy, placebo or no treatment; **O**utcome: cure rate. Secondary objectives were to assess the adverse events reported with azole treatment for TL, to verify whether responses to azole therapy are dose-dependent, species-dependent or associated with the disease geographical distribution (New and Old World), and to determine the rates of relapse and late mucosal involvement after treatment.

### Materials and methods

The review methodology was registered in the International Prospective Register of Systematic Reviews (PROSPERO; CRD42016048668; http://www.crd.york.ac.uk/PROSPERO/display_record.asp?ID=CRD42016048668), and recommendations of the Cochrane Handbook [18] and of the PRISMA statement [19] were followed. Structured searches were conducted independently by two reviewers (ELG and GFC) in EMBASE, Web of Science, PubMed (MEDLINE) and VHL (LILACS and IBECS) using a comprehensive list of key terms that were adapted to each database through October 2016 (updated July 2017). The initial search was complemented by a manual search of reference lists from retrieved articles. Furthermore, a search of gray literature (*Google Scholar*) and in clinicaltrials.gov were performed for identifying potential ongoing studies. The detailed search strategies are described in S1 File.

There were no restrictions on the publication language, date of publication or study design. The papers were included if cure rate after systemic fluconazole, itraconazole or ketoconazole therapy for TL were reported. Studies involving non-human participants, studies with less than ten patients in the azole therapy arm, and those addressing azole topical treatment or azole therapy combined with another anti-*Leishmania* active drug were excluded. Furthermore, review articles and letters to the editor were also excluded. Concomitant antibiotic use was not considered exclusion criteria. All studies matching the inclusion criteria were reviewed by the authors, and disagreements on inclusion were resolved by consensus.

After analysis of title and abstract, the selected studies were read in full to confirm their eligibility and to extract the data. The following information was recorded: country; year of publication, design of the study, predominant *Leishmania* species, therapeutic schedule, and outcomes using a standardized data collection form. The outcome of interest was clinical cure, defined as complete ulcer healing, and was assessed at three time points counted from the first day of the treatment: (1) “initial response,” assessed at 30–73 days; (2) “initial cure,” assessed at 74–100 days; and (3) “definitive cure,” assessed at 101–194 days. The intervals for the cure evaluation were an adaptation of the current recommendations for CL trials [20] to include the outcomes reported in the original studies. The final efficacy rate analysis considered the last cure rate available in the studies within six months. Relapse was assessed only for patients who were treated and were considered cured.

The quality of the randomized studies was evaluated using the following criteria: 1) double-blind; 2) concealment of treatment allocation; 3) blinding of outcome assessment; and 4) intention-to-treat analysis. Concealment of treatment allocation was considered adequate if the patients and enrolling investigators could not predict assignment. Outcome assessment was blinded if the investigator who assessed the outcome had no knowledge of treatment assignment. The analysis was performed according to the intention-to-treat principle if all randomized patients were included in the analysis and maintained in their originally assigned groups. The Newcastle-Ottawa Scale (NOS) [21] was used to assess the quality of nonrandomized studies. On this scale, the studies were measured considering three dimensions: 1) selection of study groups, 2) comparability of groups, and 3) determination of the results of interest. For randomized and non-randomized studies, it was assumed inadequate if there was not enough information to assess the quality.

### Quantitative data synthesis

Data analyses were performed using R software with “meta” and “metafor” packages, except for the meta-regression analyses that were conducted using Comprehensive Meta-Analysis, version 3. Two types of statistical analysis were performed: meta-analysis of main effects (for each one of the azole drugs in three time points) and subgroup analysis to test for effect modification by the one categorical covariate (cure rate according to therapy schedule, to *Leishmania* species, to geographical localization and adverse events). The threshold for statistical significance was 0.05. Direct comparisons between azoles and another comparator arm were performed when available. Forest plots are presented to illustrate the effects of global estimation and sub-analyses on meta-analysis results. The inconsistency (*I*^*2*^) statistic was used to evaluate heterogeneity. A random model was chosen for all analyses due to considerable heterogeneity across studies.

Considering that the association between effect and dose is evidence of the action of a drug, with the intention to identify an ideal dosage regimen, we attempted to analyze efficacy according to the azole dose used. The daily intake of azoles in milligrams was categorized as low (≤ 200 mg), intermediate (201 to 400 mg) and high (> 400 mg) dosage. A further analysis was performed considering doses categorized as less than 400 mg or equal to or greater than 400 mg. A meta-regression to determine whether the dosage could explain the variation in effect size values between studies was also performed. Studies that did not present a defined dosage were excluded from these analyses. Publication bias was assessed by observing the symmetry of funnel plots and the Begg’s adjusted rank correlation test [22].

## Results

**Fig 1:**
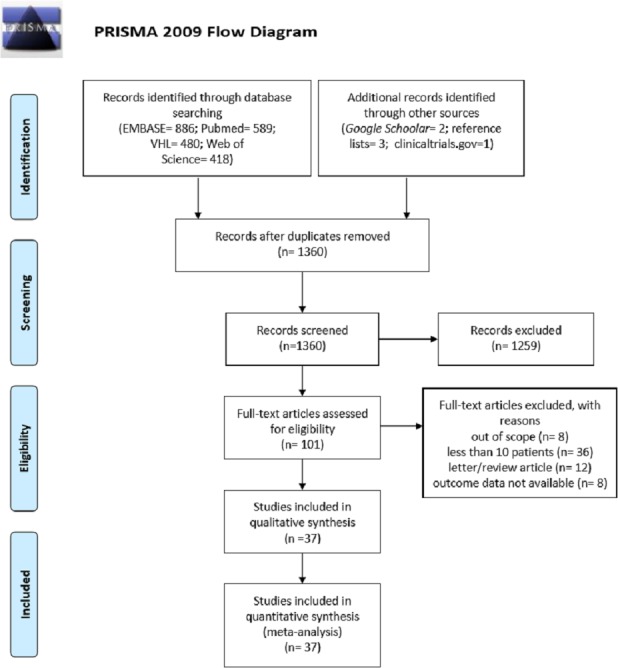
Flow diagram of the study selection process.

Our search identified 33 articles from EMBASE, Web of Science, PubMed and VHL databases. A manual search using a reference list of selected papers added two additional papers. Finally, through *Google Scholar*, a potentially relevant thesis and one article were also identified. An already published study was found on clinicaltrials.gov. Thus, 37 studies [15–17,23–56] involving 1259 patients presenting CL or ML treated with systemic fluconazole, itraconazole or ketoconazole were included. Three studies evaluated only patients presenting mucosal leishmaniasis [40,42,49]. The process of study selection and reasons for exclusion are summarized in a PRISMA flow diagram (Fig 1).

After numerous attempts, we were unable to obtain the Saleem *et al*. (2007) study in its entirety [47]. Thus, for this study, only the data available in the abstract were used, so these results were included in the meta-analysis but not in all tables that detailed the characteristics of the studies.

Among the 37 studies included, 18 were prospective non-randomized studies, 5 were retrospective studies and 14 were randomized clinical trials (RCT). Of these, 24 studies were conducted in the Old World and 13 in the New World. A study depicting the evolution of American soldiers infected during the Iraq war was allocated along with Old World studies due to the *Leishmania* species involved (*L*. *major*) [44].

The cure criteria defined by the authors varied widely among studies, and eight studies did not report the criteria used [23–26,44,50,53,55]. Most studies defined cure as complete re-epithelialization of all lesions. However, in one study, cure was defined by “*Leishmania donovani* bodies negativity” [29], while another considered “reduction in the size of lesions by 80% up to complete clearance” [26], and a third considered “more than 90% re-epithelization and negative smear for *Leishmania* parasites” [33]. Systemic therapeutic regimens with azoles encompassed the use of itraconazole, ketoconazole or fluconazole. Fifteen studies evaluated itraconazole, fourteen evaluated ketoconazole, and nine studies addressed fluconazole therapy. The main characteristics of studies, namely, inclusion, exclusion and cure criteria, are presented in Tables 1 and 2.

**Table 1:** Main methodological characteristics of the Old World leishmaniasis studies.

Year, Author	Country (cases)	Study arms (patients)	Prospective/ Comparative	Randomized	Inclusion criteria	Exclusion criteria	Cure criteria	Follow-up (months)
**1987, Zahaf**	Tunisia (10)	Ketoconazole (10)	Yes/No	No	Clinical diagnosis or parasitologically confirmed CL	NR	Complete re-epithelialization and negative direct skin smear	1,5
**1990, Dogra**	India (20)	Itraconazole (15)	Yes/ Yes	Yes	Parasitologically confirmed CL	NR	Complete re-epithelialization and negative direct skin smear	3
		No treatment (5)						
**1991, Al-Fouzan**	Kuwait (24)	Itraconazole (15)	Yes/ Yes	Yes	Parasitologically confirmed CL	NR	NR	3
		Placebo (9)						
**1992, Norton**	Egypt/ Israel (23)	Ketoconazole (23)	No/No	No	Clinical diagnosis or parasitologically confirmed CL	NR	Reduction in the size of lesion by 80% up to complete clearance	8
**1993, Singh**	India (30)	Ketoconazole (30)	Yes/ Yes	No	Parasitologically confirmed CL due to *Leishmania donovani*	Pregnancy, children below 2 years	Negative direct skin smear	1,5
		No treatment (10)						
**1994, Dogra**	India (20)	Itraconazole (10)	Yes/ Yes	Yes	Parasitologically confirmed CL	Pregnancy, children, previous antileishmanial therapy	Complete re-epithelialization and negative direct skin smear	6
		No treatment (10)						
**1994, Enden**	Belgium (22)	Itraconazole (22)	Yes/No	No	Parasitologically confirmed CL, absence/failure of previous other specific antileishmanial treatment, absence of spontaneous healing tendency, informed consent obtained.	Pregnancy	Complete re-epithelialization	6
**1995, Alsaleh**	Kuwait (33)	Ketoconazole (18)^a^	Yes/ Yes	No	Parasitologically confirmed CL	Pregnancy, age younger than 14 years, nursing women	More than 90% re-epithelization and negative direct skin smear	6
		Ketoconazole (15)^b^						
**1995, Singh**	India (16)	Ketoconazole (16)	Yes/No	No	Parasitologically confirmed CL due to *Leishmania tropica*.	Previous antileishmanial therapy	Complete re-epithelialization and, no evidence of inflammation, negative direct skin smear	2
**1996, Dogra**	India (20)	Itraconazole (10)	Yes/ Yes	Yes	Parasitologically confirmed CL	Chronic disease, abnormality in liver function tests, children below 18 years immunocompromised, previous antileishmanial therapy, lactating females	Complete re-epithelialization accompanied by 3 consecutive and negative direct skin smear	3
		Placebo (10)						
**1996, Momemi**	Iran (140)	Itraconazole (65)	Yes/ Yes	Yes	Parasitologically confirmed CL	Pregnancy, age younger than 12 years, duration of disease of more than 4 months.	Complete re-epithelialization and negative direct skin smear	1
		Placebo (66)						
**1997, Ozgoztasi**	Turkey (72)	Ketoconazole (32)	Yes/ Yes	Yes	NR	Pregnancy, nursing, serious concomitant diseases	Complete re-epithelialization	2
		P-ointment by El-On (40)						
**1997, Viriyavejakul**	Thailand (11)	Ketoconazole (11)	No/No	No	NR	NR	Complete re-epithelialization and/or scar formation	6
**1998, Siddiqui**	Saudi Arabia (55)	Itraconazole (37)	Yes/No	No	Ability to attend King Fahad Hospital at fortnightly intervals, absence of systemic diseases, no previous antileishmanial treatment	Pregnancy, systemic disease, inability to attend at regular intervals	Complete re-epithelialization and negative direct skin smear	1
		SSG (18)						
**2001, Salmanpour**	Iran (96)	Ketoconazole (64)	Yes/ Yes	Yes	NR	Pregnancy, lactating females, children below 3 years, systemic disease	Complete re-epithelialization at 6 weeks post-treatment.	6
		MA (32)						
**2002, Alrajhi**	Saudi Arabia (209)	Fluconazole (106)	Yes/ Yes	Yes	NR	Lesions that were not parasitologically confirmed by smear, culture, or both.	Complete re-epithelialization	12
		Placebo (103)						
**2005, Nassiri-Kashani**	Iran (200)	Itraconazole (100)	Yes/ Yes	Yes	Parasitologically confirmed CL	Pregnancy, lactating females, females in child-bearing age without contraception, receipt of antileishmanial treatment previous, allergy to itraconazole, Duration of lesions > 45 days, lesions on face or near the mucous membranes, more than 5 lesions, lesion with a diameter > 3 cm	Complete re-epithelialization	3
		Placebo (100)						
**2005, Willard**	Iraq (237)	Fluconazole (15)	No/ Yes	No	Parasitologically confirmed CL	NR	NR	6
		Itraconazole (2)						
		SSG (62)						
		Thermotherapy (26)						
		Cryotherapy (4)						
**2007, Morizot**	France/Swiss (45)	Fluconazole (45)	Yes/ No	No	Parasitologically confirmed CL, if clinical follow-up over the telephone was performed later than day 90	Pregnancy, systemic disease, acquired immunodeficiency syndrome, disease of the oronasal mucosa	Complete re-epithelialization	3
**2007, Rafaa**	France (14)	Fluconazole (14)	Yes/No	No	NR	NR	Complete re-epithelialization	3
**2007, Saleem**	Pakistan (200)	Itraconazole (100)	Yes/ Yes	No				
		MA (100)						
**2009, Al-Mutairi**	Kuwait (78)	Itraconazole (12)	No/ Yes	No	NR	NR	Complete re-epithelialization, no relapse during 6 months after completion of therapy, negative direct skin smear	6
		Cryotherapy (44)						
		Dapsone (16)						
		Imiquimod (6)						
**2011, Emad**	Iran (120)	Fluconazole (60)^c^	Yes/ Yes	Yes	NR	NR	NR	1,5
		Fluconazole (60)^d^						
**2014, Khan**	Saudi Arabia (20)	Fluconazole (10)	Yes/Yes	No	NR	NR	NR	8
		Itraconazole (10)						

**NR:** not reported; **CL:** cutaneous leishmaniasis; **SSG**: Sodium stibogluconate; **MA**: Meglumine antimoniate

^a^: 600 mg/once

^b^: 800 mg/once

^c:^ 100 mg/twice

^d^: 200 mg/twice

**Table 2:** Main methodological characteristics of the New World leishmaniasis studies.

Year, Author	Country (cases)	Study arms (number of patients)	Prospective/ Comparative	Randomized	Inclusion criteria	Exclusion criteria	Cure criteria	Follow-up (months)
**1986, Dedet**	Suriname (12)	Ketoconazole (12)	Yes/No	No	Parasitologically confirmed CL	NR	NR	2
**1987, Restrepo**	Colombia (16)	Ketoconazole (12)	Yes/ Yes	No	Parasitologically confirmed CL	Clinical cure not achieved within 60 days of treatment	NR	1
		Ketoconazole + cream (4)						
**1988, Scorza**	Venezuela (38)	Ketoconazole (38)	Yes/No	No	Parasitologically confirmed CL	NR	NR	12
**1988, Santos**	Brazil (21)	Ketoconazole (21)	Yes/ Yes	No	Parasitologically confirmed CL	NR	NR	24
		MA (21)						
**1990, Saenz**	Panama (41)	Ketoconazole (22)	Yes/ Yes	Yes	Parasitologically confirmed CL	Systemic disease, facial or mucosal lesions, abnormalities on baseline tests	Lesion not clinically relapsed by the 12- month follow-up examination.	12
		SSG (19)						
		Placebo (11)						
**1992, Navin**	Guatemala (120)	Ketoconazole (40)	Yes/ Yes	Yes	Parasitologically confirmed CL	No previous treatment with antimonials or imidazoles, no serious concomitant medical problems, availability for follow-up for 12 months, and no visible evidence of mucosal involvement	Complete re-epithelialization and no evidence of inflammation	12
		SSG (40)						
		Placebo (40)						
**1995, Santos**	Brazil (26)	Itraconazole (26)	Yes/No	No	NR	NR	Complete re-epithelialization	9
**2000, Amato**	Brazil (10)	Itraconazole (10)	Yes/No	No	Clinical diagnosis or parasitologically confirmed ML	Pregnancy, previous antileishmanial therapy (6 months), and transaminase alterations	Complete re-epithelialization in a maximum 12 weeks after end of treatment.	3
**2004, Calvopina**	Ecuador (13)	Itraconazole (13)	Yes/No	No	Clinical diagnosis or parasitologically confirmed ML	Pregnancy, allergy to itraconazole or related drugs, serious concomitant diseases, previous antileishmanial therapy (3 months)	Complete re-epithelialization	12
**2009, Amato**	Brazil (140)	Itraconazole (15)	No/ Yes	No	NR	Contraindication to the drug under evaluation or adverse effects to the current drug	Complete re-epithelialization in a maximum 12 weeks after end of treatment.	18
		MA (73)						
		Pentamidine (22)						
		D-AmB (17)						
		AmCD (9)						
		L-AmB (4)						
**2011, Sousa**	Brazil (28)	Fluconazole (28)	Yes/No	No	Parasitologically confirmed CL	Pregnancy, systemic disease, lactating females	Complete re-epithelialization	1
**2012, Silva**	Brazil (120)	Fluconazole (60)	Yes/ Yes	Yes	Parasitologically confirmed CL	Pregnancy, age < 18 years, cardiac, renal, or liver disease, acquired immunodeficiency syndrome, previous ML, no previous antileishmanial treatment before enrolment	Complete re-epithelialization and no evidence of inflammation	3
		MA (60)						
**2016, Prates**	Brazil (53)	Fluconazole (27)	Yes/Yes	Yes	Parasitologically confirmed CL, illness duration >1 month and < 3 months, age 18–65 years, 1–3 ulcerated lesions, and major ulcer diameter ranging from 10 to 50 mm	Pregnancy, lactating females, severe disease, allergy to fluconazole or MA, uncontrolled active infectious	Complete re-epithelialization and no evidence of inflammation	6
		MA (26)						

**NR:** not reported; C**L:** cutaneous leishmaniasis; **ML:** mucosal leishmaniasis; **MA:** meglumine antimoniate; M**A-IL:** intralesional meglumine antimoniate; **SSG:** sodium stibogluconate; **Penta120-IL:** intralesional pentamidine; **L-AmB:** liposomal amphotericin B; **AmCD:** Amphotericin colloidal dispersion; **D-AmB**: Deoxycholate anphotericin B

The population we gathered included young adults, and only one study included children exclusively [46]. In general, the mean number of lesions per patient with regard to men:women ratio and time of disease were higher in Old World studies compared to those in the New World (Tables 3 and 4). The mean follow-up time was relatively short (maximum 24 months), and the late mucosal involvement rate was not evaluated in studies.

**Table 3:** Characteristic of the population enrolled in azole arm in the Old World leishmaniasis studies.

Year, Author	Age (mean, years)	Gender male/female	Mean of lesions per patient	Mean of lesion area/mm^2^	Mean of lesion duration (weeks before therapy)	*Leishmania* species characterization (n/n total)
**1987, Zahaf**	35.0	NR	2.7	NR	1.5	NR
**1990, Dogra**	34.0	11/4	1.8	NR	8.8	NR
**1991, Al-Fouzan**	NR	13/11	NR	NR	NR	NR
**1992, Norton**	NR	25/0	NR	NR	NR	NR
**1993, Singh**	NR	17/13	NR	NR	NR	*L*. *donovani* (30/30)
**1994, Dogra**	NR	NR	NR	NR	NR	NR
**1994, Enden**	34.4	16/6	NR	NR	19.6	NR
**1995, Alsaleh**	36.0	25/8	3.42	NR	5.3	NR
**1995, Singh**	NR	11/5	NR	NR	NR	*L*. *tropica* (16/16)
**1996, Dogra**	NR	NR	NR	NR	NR	NR
**1996, Momemi**	26.0	44/21	3.3	NR	5.4	NR
**1997, Ozgoztasi**	NR	NR	NR	NR	NR	NR
**1997, Viriyavejakul**	34.3	11/0	2.4	NR	3.6	NR
**1998, Siddiqui**	36.2	30/5	2.9	NR	2.3	NR
**2001, Salmanpour**	20.7	30/34	2.5	NR	2.6	NR
**2002, Alrajhi**	31.2	208/1	3.1	17	9.2	NR
**2005, Nassiri-Kashani**	NR	NR	2.5	7.76	NR	NR
**2005, Willard**	NR	NR	NR	NR	NR	NR
**2007, Morizot**	41.0	16/19	4.0	NR	4.5	*L*. *major* (27/45)
						*L*. *tropica* or *L*. *infantum* or both (8/45)
**2007, Rafaa**	6.0	7/7	2.8	NR	NR	NR
**2009, Al-Mutairi**	NR	NR	NR	NR	NR	NR
**2011, Emad**	35.9	65/55	3.1	20.8	NR	NR
**2014, Khan**	NR	19/8	NR	NR	NR	*L*. *major* (17/27)
						*L*. *tropica* (10/27)

**NR:** not reported

**Table 4:** Characteristic of the population enrolled in the azole arm in the New World leishmaniasis studies.

Year, Author	Age (mean, years)	Gender male/female	Mean of lesions per patient	Mean of lesion area/mm^2^	Mean of lesion duration (weeks before therapy)	*Leishmania* species characterization (n/n total)
**1986, Dedet**	27.6	11/1	2.41	NR	5.1	*L*. *braziliensis* (11/12)
						*L*. *mexicana* (1/12)
**1987, Restrepo**	NR	NR	NR	NR	NR	NR
**1988, Scorza**	26.5	12/26	1.68	NR	NR	NR
**1988, Santos**	16.0	15/6	1.61	NR	3.09	NR
**1990, Saenz**	25.0	22/0	2.1	333.0	8.2	NR
**1992, Navin**	20.2	NR	1.5	220.0	9.7	*L*. *braziliensis* (23/120)
						*L*. *mexicana* (32/120)
**1995, Santos**	NR	26/0	NR	NR	NR	*L*. *braziliensis (*26/26)
**2000, Amato**	54.6	NR	NR	NR	NR	NR
**2004, Calvopina**	42.0	11/2	NR	NR	144	*L*. *braziliensis (*2/13)
**2009, Amato**	65.0	10/5	NR	NR	NR	NR
**2011, Sousa**	37.5	13/16	NR	NR	10.0	NR
**2012, Silva**	41.0	26/34	1.4	NR	4.9	*L*. *braziliensis (*60/60)
**2016, Prates**	27.9	15/12	1.2	270.6	NR	NR

**NR:** not reported

The concomitant use of antibacterial therapy was mentioned only by two studies [17,55] intending to treat lesions with secondary bacterial infection. The azole therapeutic regimens varied significantly among the studies. The daily dose ranged from 100 to 800 mg, and the treatment duration ranged from 14 to 84 days. The mean treatment length was similar in Old (40.3 days) and New World studies (45.5 days). The therapeutic schedules are presented in Tables 5 and 6.

**Table 5:** Azole therapy schedules among Old World leishmaniasis studies.

Year, (Author)	Country	Azole agent (number of patients treated)	Daily dosage (mg/frequency)	Treatment length (days)
**1987, Zahaf**	Tunisia	KTZ (10)	400 mg/twice	21–42
			5 drops/kg (children)	
**1990, Dogra**	India	ITCZ (15)	Maximum 200 mg/once	42
**1991, Al-Fouzan**	Kuwait	ITCZ (15)	100 mg/twice	42–56
			3 mg/ kg/ once (children)	
**1992, Norton**	Egypt/ Israel	KTZ (23)	200 mg/ once	30
**1993, Singh**	India	KTZ (30)	400 mg/ once	14–40
**1994, Dogra**	India	ITCZ (20)	Maximum 200 mg/ once	42
**1994, Enden**	Belgium	ITCZ (22)	100 mg/ twice	28–56
			5 mg/kg/ once (children)	
**1995, Alsaleh**	Kuwait	KTZ (18)	600 mg/ once	42
**1995, Alsaleh**	Kuwait	KTZ (15)	800 mg/once	42
**1995, Singh**	India	KTZ (16)	200 mg/ twice	70
**1996, Dogra**	India	ITCZ (10)	100 mg/ twice	42
**1996, Momemi**	Iran	ITCZ (65)	Maximum 400 mg/ once	21
**1997, Ozgoztasi**	Turkey	KTZ (32)	400 mg/ once	30
			200 mg/ once (children)	
**1997, Viriyavejakul**	Thailand	KTZ (11)	400 mg/ once	28
**1998, Siddiqui**	Saudi Arabia	ITCZ (37)	400 mg/ once	30
**2001, Salmanpour**	Iran	KTZ (64)	600 mg/ once	30
			10 mg/kg/once (children)	
**2002, Alrajhi**	Saudi Arabia	FCZ (106)	200 mg/ once	42
**2005, Nassiri-Kashani**	Iran	ITCZ (100)	200 mg/ once	56
**2005, Willard**	Iraq	FCZ (15)	200 mg/ twice	42
**2007, Morizot**	France/Swiss	FCZ (45)	200 mg/ once	42
			2.5 mg/kg/ once (children)	
**2007, Rafaa**	France	FCZ (14)	2.5 mg/kg/ once	42
**2007, Saleem**	Pakistan	ITCZ (100)	100 mg/ twice	42–56
**2009, Al-Mutairi**	Kuwait	ITCZ (12)	100 mg/ twice	42–56
**2011, Emad**	Iran	FCZ (60)	100 mg/twice	42
**2011, Emad**	Iran	FCZ (60)	200 mg/twice	42
**2014, Khan**	Saudi Arabia	FCZ (10)	200 mg/once	42
**2014, Khan**	Saudi Arabia	ITCZ (10)	150 mg/ once	42

**ITCZ:** itraconazole; **FCZ:** fluconazole; **KTZ:** ketoconazole

**Table 6:** Azole therapy schedules among New World leishmaniasis studies.

Year, (Author)	Country	Azole agent (number of patients treated)	Daily dosage (mg/frequency)	Treatment length (days)
**1986, Dedet**	French Guiana/Suriname	KTZ (12)	400 mg/ once	30
**1987, Restrepo**	Colombia	KTZ (12)	400 mg/ once	60
**1988, Scorza**	Venezuela	KTZ (38)	100–200 mg/ twice	NR
			200 mg/ once (children)	
**1988, Santos**	Brazil	KTZ (21)	5–10 mg/kg/ once	60
**1990, Saenz**	Panama	KTZ (22)	600 mg/ once	28
**1992, Navin**	Guatemala	KTZ (40)	600 mg/ once	28
**1995, Santos**	Brazil	ITCZ (26)	100 mg/ once	60
**2000, Amato**	Brazil	ITCZ (10)	4 mg/kg per day/ twice	42
**2004, Calvopina**	Ecuador	ITCZ (13)	400 mg/ twice	84
**2009, Amato**	Brazil	ITCZ (10)	200 mg/ once	42
**2011, Sousa**	Brazil	FCZ (28)	5–8 mg/kg/ once	42
**2012, Silva**	Brazil	FCZ (60)	300–450 mg/ once	42
**2016, Prates**	Brazil	FCZ (27)	6.5–8 mg/ once	28

**NR:** not reported; **ITCZ:** itraconazole; **FCZ:** fluconazole; **KTZ:** ketoconazole

Only nine studies (24%) reported *Leishmania* species characterization [28,29,32,34,42,45,52,53,55]. *L*. *braziliensis* was the species more prevalent in the Americas, while *L*. *tropica* and *L*. *major* were usually reported in the Old World. The summarized cure rates according to the intention-to-treat analysis and adverse events rates are shown in Tables 7 and 8.

**Table 7:** Outcome in Old World leishmaniasis studies.

Year, Author	Azole agent (number of treated patients)	Epithelialization rate between 30–73 days, cured/treated patients (%)	Epithelialization rate between 74–100, cured/treated patients (%)	Epithelialization rate between 101–194 days, cured/treated (%)	Follow-up lost lost/treated patients	Number of patients with adverse events /total of patients treated (%)	Relapse after cure /number of patients cured
**1987, Zahaf**	KTZ (10)	8/10 (0.8)	NR	NR	0/10	1/10 (10)	NR
**1990, Dogra**	ITCZ (15)	10/15 (66.6)	NR	NR	0/15	3/15 (20)	0/10 (0)
**1991, Al-Fouzan**	ITCZ (15)	11/15 (73.3)	NR	NR	0/15	3/15 (20)	0/11 (0)
**1992, Norton**	KTZ (23)	22/23 (96)	NR	NR	0/23	NR	0/22 (0)
**1993, Singh**	KTZ (30)	28/30 (93.3)	NR	NR	0/30	1/30 (3.3)	NR
**1994, Dogra**	ITCZ (20)	15/20 (75)	NR	NR	0/20	3/20 (15)	0/15 (0)
**1994, Enden**	ITCZ (22)	15/22 (68.1)	NR	17/22 (77.2)	3/22	5/19 (26.3)	1/17 (0.06)
**1995, Alsaleh**	KTZ (18)^a^	12/18 (66.6)	NR	NR	3/18	1/18 (5.5)	0/12 (0)
**1995, Alsaleh**	KTZ (15)^b^	9/15 (60)	NR	NR	4/15	1/15 (6.6)	0/9 (0)
**1995, Singh**	KTZ (16)	0/16 (0)	0/16 (0)	NR	2/16	NR	NA
**1996, Dogra**	ITCZ (10)	7/10 (70)	NR	NR	0/10	2/10 (20)	NR
**1996, Momemi**	ITCZ (65)	36/65 (55.4)	NR	NR	4/65	6/61 (9.8)	NR
**1997, Ozgoztasi**	KTZ (32)	0/32 (0)	NR	NR	0/32	0/32 (0)	NA
**1997, Viriyavejakul**	KTZ (11)	9/11 (81.8)	8/11 (72.7)	7/11 (63.6)	0/11	0/11 (0)	2/9 (22.2)
**1998, Siddiqui**	ITCZ (37)	22/37 (59.4)	NR	NR	2/37	0/37 (0)	NR
**2001, Salmanpour**	KTZ (64)	57/64 (89)	NR	NR	0/64	NR	NR
**2002, Alrajhi**	FCZ (106)	23/106 (21.6)	63/106 (59.4)	NR	26/106	NR	0/63 (0)
**2005, Nassiri-Kashani**	ITCZ (100)	49/100 (49)	67/100 (67)	NR	17/100	4/83 (4.8)	NR
**2005, Willard**	FCZ (15)	11 /15 (78.5)	NR	NR	0/15	1/15 (6.6)	NR
**2007, Morizot**	FCZ (45)	15/45 (33.3)	NR	NR	10/45	4/35 (11.4)	NR
**2007, Rafaa**	FCZ (14)	5/14 (36)	10/14 (71)	NR	0/14	1/14 (7.1)	NR
**2009, Al-Mutairi**	ITCZ (12)	3/12 (25)	NR	NR	0/12	NR	NR
**2011, Emad**	FCZ (60)^c^	29/60 (48.3)	NR	NR	0/60	0/60	NR
**2011, Emad**	FCZ (60)^d^	47/60 (78.3)	NR	NR	2/60	NR	NR
**2014, Khan**	FCZ (10)	7/10 (70)	NR	NR	0/10	2/10 (20)	NR
**2014, Khan**	ITCZ (10)	6/10 (60)	NR	NR	0/10	1/10 (10)	NR

**NR:** not reported; **NA:** not applicable; **ITCZ:** itraconazole; **FCZ:** fluconazole; **KTZ:** ketoconazole

^a^: 600 mg/once

^b^: 800 mg/once

^c:^ 100 mg/twice

^d^: 200 mg/twice

**Table 8:** Outcomes in New World leishmaniasis studies.

Year, Author	Azole agent (number of patients)	Epithelialization rate between 30–73 days, cured/treated patients (%)	Epithelialization rate between 74–100, cured/treated patients (%)	Epithelialization rate between 101–194 days, cured/treated patients (%)	Follow-up lost lost/treated patients	Number of patients with adverse events /total of patients treated (%)	Relapse after cure /number of patients cured
**1986, Dedet**	KTZ (12)	1/12 (8.3)	3/12 (25)	NA	0/12	9/12 (75.0%)	NR
**1987, Restrepo**	KTZ (12)	1/12 (8.3)	4/12 (33.3)	NR	5/12	0/12	NR
**1988, Scorza**	KTZ (38)	6/38 (15.7)	29/38 (76.2)	38/38 (100)	0/38	NR	2/38 (0.05)
**1988, Santos**	KTZ (21)	18/21 (85.7)	NR	NR	0/21	3/21 (15.7)	1/21 (0.05)
**1990, Saenz**	KTZ (22)	7/22 (31.8)	16/22 (72.7)	16/22 (72.7)	0/22	12/22 (54.5)	2/16 (12.5)
**1992, Navin**	KTZ (40)	2/40 (5)	12/40 (30)	10/40 (25)	2/40	7/40 (17.5)	2/12 (16.6)
**1995, Santos**	ITCZ (26)	25/26 (96.1)	26/26 (100)	NA	0/26	NR	0/26 (0)
**2000, Amato**	ITCZ (10)	6/10 (60)	NR	NR	0/10	0/10 (0)	NR
**2004, Calvopina**	ITCZ (13)	NR	3/13 (23)	0/10 (0)	0/13	0/13 (0)	NR
**2009, Amato**	ITCZ (15)	11/15 (73.3)	NR	NR	0/15	NR	2/11 (18)
**2011, Sousa**	FCZ (28)	NR	25/28 (89.2)	NR	0/28	1/28 (3.5)	NR
**2012, Silva**	FCZ (60)	40/60 (66.6)	NA	NA	0/60	0/60	NR
**2016, Prates**	FCZ (27)	6/27 (22.2)	NR	6/27 (22.2)	0/27	NR	0/6 (0)

**NR:** not reported; **NA:** not applicable; I**TCZ:** itraconazole; **FCZ:** fluconazole; **KTZ:** ketoconazole

The relapse rate after cure was reported in only 43% of studies. Among 282 patients cured in these studies, 10 (4%) relapsed. Only two studies (5.4%) did not mention the occurrence of side effects. However, in general, side effects were poorly described. Many authors reported only the absence of serious events leading to treatment interruption. Adverse events that were reported as reasons to discontinuation of azole therapy were increased liver enzymes, epigastric pain, nausea and vomiting, increased creatinine, headaches, skin rash, and jaundice. An overview of adverse events reported by the authors of included studies are reported in S1 and S2 Tables.

### Methodological quality

According to the score system adopted for RCT quality assessment (S3 Table), the most compromised domain was related to blindness, and the least compromised was related to intention-to-treat analysis. The methodological quality assessment of the 22 non-randomized studies using Newcastle-Ottawa Scale is presented in S4 Table. Overall, studies presented low methodological quality, and none of the studies obtained the maximum score corresponding to nine stars. The risk of bias across studies for a given outcome is presented in S5 Table.

No evidence of publication bias was detected in the meta-analysis of initial response for fluconazole (z = 0.29, p = 0.79), ketoconazole (z = -0.45, p = 0.65) and itraconazole (z = -0.05, p = 0.96) or for meta-analysis of final efficacy rate for these same azoles, namely, fluconazole (z = 0.27, p = 0.79), ketoconazole (z = 0.33, p = 0.74) and itraconazole (z = -0.75, p = 0.45). For final efficacy rate from meta-analysis for Old World and New World, there was also no indication of publication bias (z = 0.02, p = 0.98 and z = 0.85, p = 0.39, respectively). Publication bias analyses were not performed for the other comparisons because of the small number of studies. Visual inspections of funnel plots did not show any substantial asymmetry (S1, S2 and S3 Figs).

### Global analysis

In a global analysis including studies addressing all azole medicines, regardless of their origin, the initial response rate was 53% (CI95%: 43–62%, I^2^ = 85.3%) and the final efficacy rate was 64% (CI95%: 57–70%, I^2^ = 78%). If only RCT studies were considered, final efficacy rate, the only outcome that could be calculated, was 60% (CI95%: 50–70%, I^2^ = 82%), which means no difference between RCT and non-RCT studies (p = 0.41). No significant difference was observed in the final efficacy rate (of all azoles and each azole group) of studies shared according to the methodological quality. There were three studies exclusively involving patients with mucosal leishmaniasis. All of them were conducted in the New World and addressed itraconazole therapy. The final efficacy rate observed for these 38 patients was 52% (CI95%: 23–79%, I^2^ = 69.5%).

**Fig 2:**
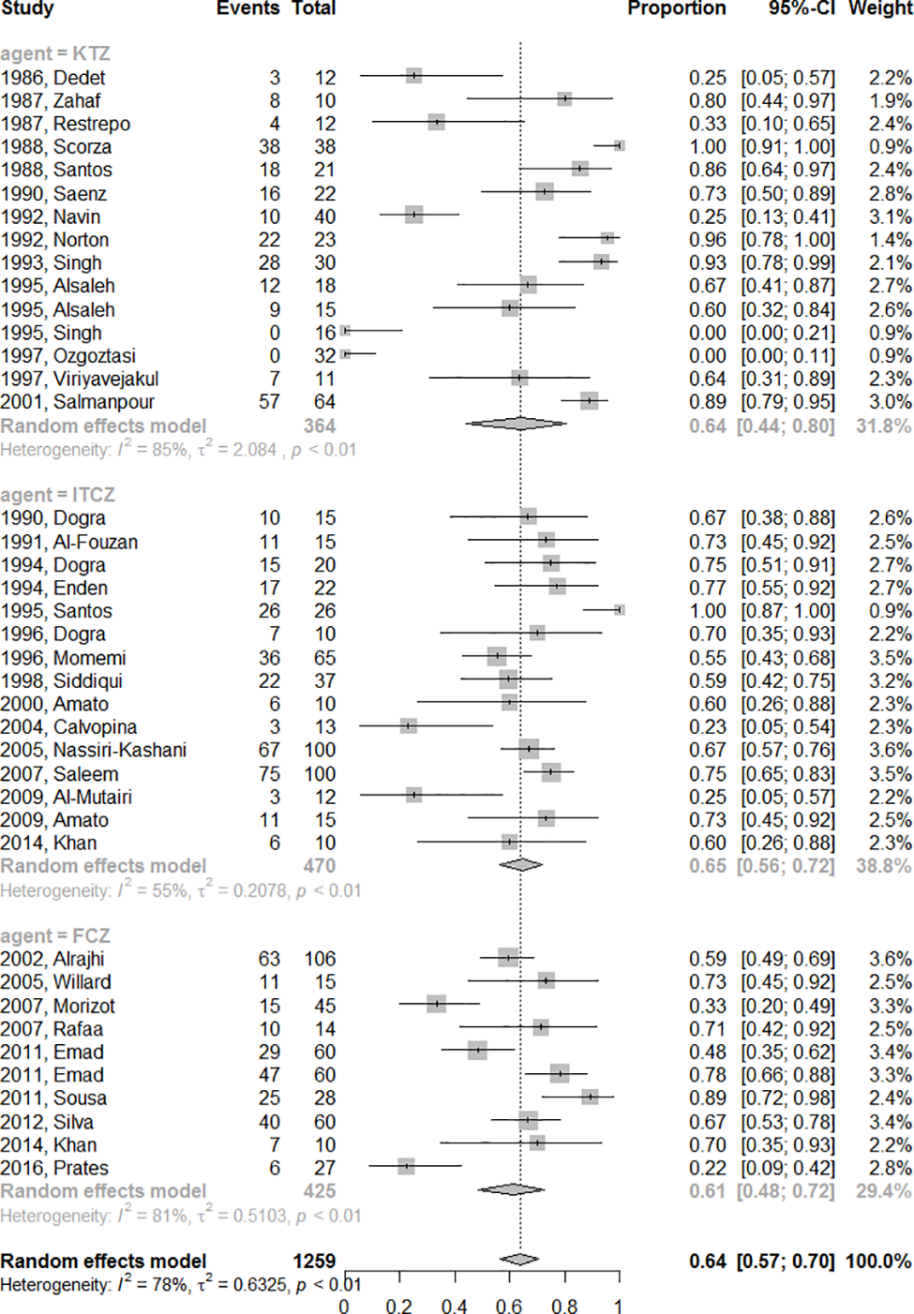
The pooled final efficacy rate of azole therapy.

In the subgroup analysis, the initial response rates did not differ according to the azole used (p = 0.89) and were 47% (CI95%: 24–70%, I^2^ = 88.7%), 45% (CI95%: 29–62%, I^2^ = 88.2%) and 61.8% (CI95%: 51–71%, I^2^ = 70.4%) for the ketoconazole, fluconazole and itraconazole groups, respectively. After exclusion of studies assessing cure by criteria other than complete re-epithelization [26,29,33], the estimated initial response rate for all azoles was 50% (CI95%: 40–60%, I^2^ = 85.7%) and the cure rates remained similar in the analysis of each azole group: 37% (CI95%: 14–67%, I^2^ = 90%) for ketoconazole, 61% (CI95%: 50–70%, I^2^ = 71.8%) for itraconazole and the same rate fluconazole (45%, CI95%: 29–62%, I^2^ = 88.2%). The final efficacy rate considering all studies were similar among the fluconazole (61%, CI95%: 48–72%, I^2^ = 81%), itraconazole (65%, CI95%: 56–72%, I^2^ = 55%) and ketoconazole (64%, CI95%: 44–80%, I^2^ = 85%) group arms (p = 0.89) (Fig 2).

**Fig 3:**
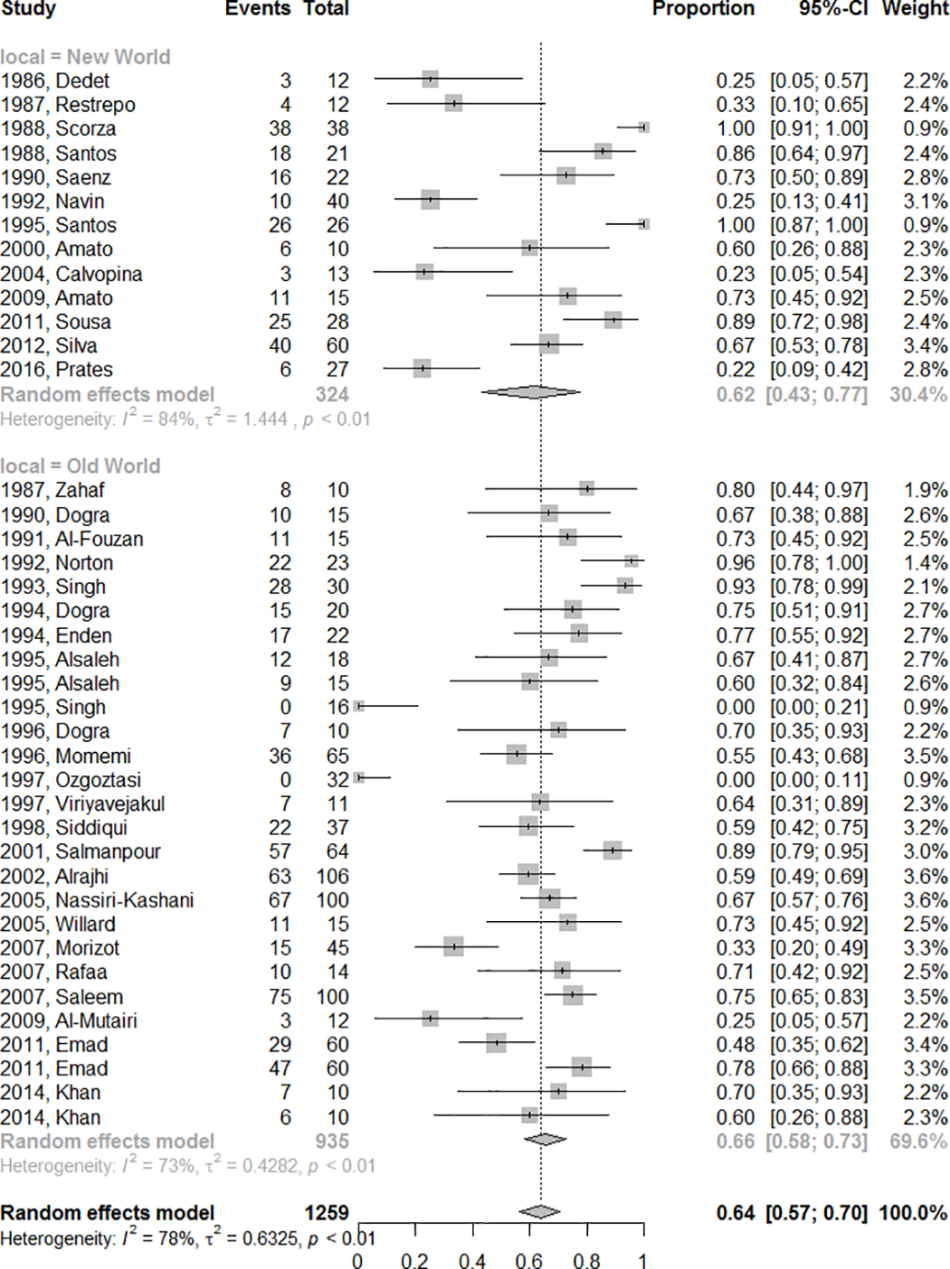
Final efficacy rate according to disease geographical distribution (Old World or New World).

The final efficacy rate for all azoles medicines in the New or Old World are presented in Fig 3, without difference between the two regions (p = 0.68).

**Fig 4:**
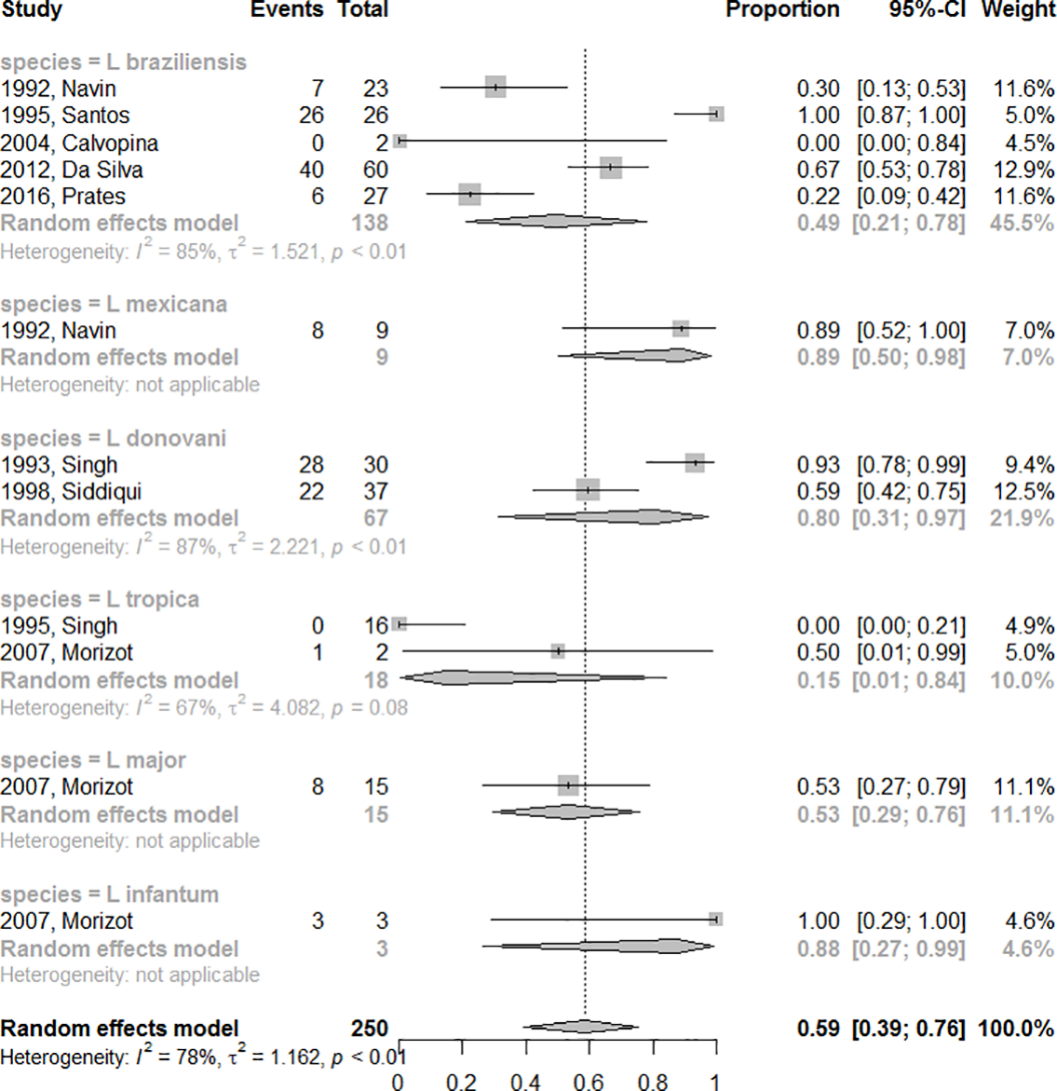
Final efficacy rate according to *Leishmania* species.

The final efficacy rate of azoles according to species are 89% for *L*. *mexicana* (CI95%: 50–98%), 88% for *L*. *infantum* (CI95%: 27–99%); 80% for *L*. *donovani* (CI%:31–97%); 53% for *L*. *major* (CI95%: 29–76%); 49% for *L*. *braziliensis* (CI95%: 21–78%) and 15% (CI95%: 1–84%) for *L*. *tropica*. This information is compiled in Fig 4.

No stratification of the patients per dose administered of the drugs, whether division into high-, intermediate- and low-dose groups (p = 0.44) or binary division with a 400 mg cutoff point (p = 0.72), allowed for detection of difference between the groups. In meta-regression analysis, no statistically significant differences across dosages were associated with effect size of azoles in general (S4 Fig). In turn, meta-regression for each of the azoles separately indicated a correlation between dose and effect with fluconazole treatment (p = 0.04) (S5 Fig).

The overall adverse events rate reported was 11% (CI95%: 7–17%, I^2^ = 64%). The adverse events rates according to azole medicine were 7% (CI95%: 3–14%) with fluconazole and 12% (CI95%: 8–19%) and 13% (CI95%: 6–29%) with itraconazole and ketoconazole, respectively, without difference among them (p = 0.35).

**Fig 5:**
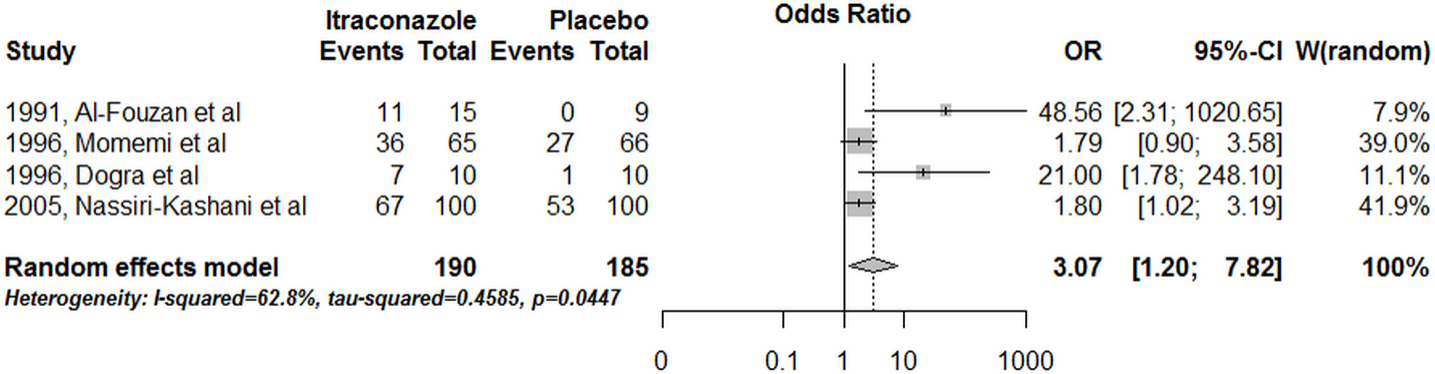
Itraconazole *versus* placebo for TL treatment in studies conducted in the Old World.

In general, the studies compared different interventions; thus, few direct comparisons were possible. Final efficacy rate using itraconazole was significantly higher compared to a placebo group in studies conducted in the Old World (OR = 3.07, CI95%: 1.2–7.8) (Fig 5).

**Fig 6:**
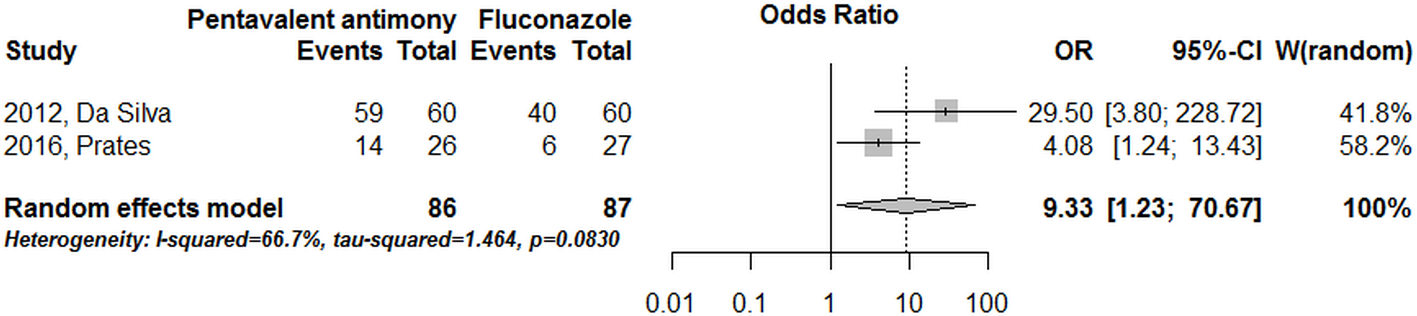
Fluconazole directly compared with systemic pentavalent antimony, in studies conducted in the New World.

Only three studies compared azoles with systemic pentavalent antimony [25,52,54], although some of them were assessed by an indirect historical comparison [25]. Meta-analysis including only studies presenting direct comparison revealed a systemic pentavalent antimony efficacy higher (88%) than that observed with fluconazole (44%) (OR = 9.33, CI 95%: 1.23–70.67) (Fig 6).

## Discussion

No previous systematic review have specifically addressed our research questions. The present study, which was based on a total population of 1259 patients, identified an overall efficacy rate of 64% (95% CI: 57–70%) in the treatment of TL with azole drugs. This compiled efficacy rate required a critical interpretation by considering all analyzes of subgroups we have done in order to overcome the limitations imposed by the high heterogeneity between studies context. Taking this important observation into account, it is possible to draw a parallel between this result and the efficacy obtained using the gold standard drug in the treatment of the disease: antimony derivatives. The efficacy rate of this first line therapy is approximately 76.5%, according to studies gathered in a systematic review for the New World [6]. Corroborating the same interpretation, an analysis of the two studies, that directly compared antimony derivatives with azoles, specifically with fluconazole, confirms the superior efficacy of the former in the treatment of American tegumentary leishmaniasis (OR = 9.33), despite the heterogeneity across the studies and the large confidence interval found.

Even with a cure rate no higher than 80% in large studies, antimony derivatives continue to be the first-line treatment recommended for all forms of leishmaniasis in many countries. In addition to a suboptimal efficacy, antimony treatment has additional disadvantages including serious adverse effects [10] and the need for long-term parenteral use and laboratory and cardiac monitoring during treatment. Therefore, the identification of new therapeutic approaches for leishmaniasis is considered a priority, and oral medication emerges as an attractive option. In addition to miltefosine, which is already included in recommendations for the treatment of TL [11], allopurinol, macrolides and various azoles have been described for the treatment of the disease. With use first proposed in the 1980s [43], fluconazole, ketoconazole and itraconazole have since been indicated for the treatment of TL in many, generally small studies of poor methodological quality [15–17,23–56]. In addition, many case reports and series with fewer than ten patients that explored the efficacy and safety of this class of medications have also been published [57–62]. In this context, most of the studies included in this systematic review were not randomized. Nonetheless, our results reveal no significant differences between the cure rates found in the randomized and non-randomized studies.

The anti-leishmanial effects of azole antifungal agents are associated with the inhibition of cytochrome P-450 mediated 14α-demethylation of lanosterol in fungi, which blocks ergosterol synthesis and causes the accumulation of 14α-methyl sterols. The inhibition of sterol biosynthesis is associated with the inhibition of leishmaniasis growth [12]. In vitro studies addressing the effects of azoles on sterol biosynthesis have revealed that, for most *Leishmania* strains, itraconazole was slightly more inhibitory than ketoconazole, and fluconazole was much less inhibitory than the other azoles [13]. In this review, it was not possible to observe differences in efficacy among the different azoles, namely, itraconazole (65%), ketoconazole (64%) and fluconazole (61%). Similarly, the impact of concomitant use of antibacterial therapy cannot be assessed in this review due the small number of patients undergoing this therapy and the absence of a uniform criterion for its use.

Itraconazole was the azole chosen for treatment in all studies that exclusively evaluated patients with the mucosal form of leishmaniasis [41,43,50]. Its better bioavailability and ability to achieve higher tissue concentrations compared to the other azoles, including in the mucosa, may explain this choice [63–65]. This review identified a cure rate of 52% for mucosal leishmaniasis in the Americas. However, the rate of late mucosal involvement after treatment with azoles was not assessed by the studies reviewed here, which generally presented a short follow-up period. This is a relevant concern for assessment, particularly in species prevalent in the Americas [66].

In 2010, azole drugs were identified by WHO as a treatment option for *L*. *major* and *L*. *mexicana* [11]. Considering the insufficient number of studies addressing the cure rate according to the *Leishmania* species, additional evidence is necessary to draw conclusions on the efficacy of azoles against each species of *Leishmania*. In contrast, our results do not reveal a difference in the final efficacy rate with azole therapy between the Old and New Worlds, which would be expected because of the different efficacy rates reported for the different *Leishmania* species [67,68]. Specifically, the final efficacy rate for *L*. *braziliensis*, a predominant species in the Americas, was 49%, based on an analysis involving only 138 patients. Based on only one or two studies with efficacy data for each of the other *Leishmania* species, the cure rate ranged from 15% for patients with *L*. *tropica* to 89% for *L*. *mexicana*. These observations reinforce that the actual efficacy of azoles treatment still needs to be determined and that the gathering of non-comparative and methodologically fragile studies is an imperfect strategy for understanding the usefulness of this class of drugs.

Among the studies included in this review, only one reported exclusively on the cure rate in children [46], preventing an overall summary analysis of efficacy in this age group. Future clinical trials should be conducted considering the therapeutic response related to the clinical presentation of the disease and the host response according to age to support more specific recommendations by patient subgroup.

In general, adverse events were not observed systemically and were not reported in a standardized way by the authors. Whereas some studies counted the number of events, others reported the rate of patients affected by these events. Based on most of the available data, the overall rate of individuals affected by adverse events due to azoles in this review was 11%. Despite this attempt to synthesize the rate of adverse events, our analysis included studies that did not measure this outcome or used different drugs (fluconazole, itraconazole and ketoconazole) and varied dosing regimens; thus, this result should be interpreted with caution.

The dose-effect association observed with fluconazole can be understood, in addition to evidence of the action of the drug, as an indication that the full efficacy may not yet have been achieved with the doses assessed. In summary, our results for the pooled overall efficacy rate of azoles reveal the fragility of this analysis strategy but, on the other hand, although modest, are corroborated by the direct comparison performed between itraconazole *versus* placebo (OR = 3.07, 375 patients), what could be taken as an indicative of activity of this class of drugs in the treatment of TL. The identified activity and dose-effect association reinforces the need for further studies evaluating the efficacy and safety of azoles at higher doses, particularly in the New World, where the rate of spontaneous cure is low [68] and the available data suggest a reasonable efficacy against *L*. *mexicana*.

The main limitation of this review is the low quality of the available studies. Many studies were conducted at a time prior to the standardization of criteria for clinical studies [69], when the requirements for the performance and reporting of methods were less strict. This systematic review included studies with small samples and both non-comparative and non-randomized studies; moreover, only four studies performed a sample size calculation [17,36,43,54]. The heterogeneity found here is evident in the various treatment schemes with the azoles and *Leishmania* species involved. Assessing the overall risk of bias for a given outcome across studies, a high risk of bias was observed in the comparison of efficacy between the different azoles, mainly due to differences in the cure criteria adopted by the groups. Related to the same domain (cure criteria), a moderate risk of bias was observed in the comparison between endemic regions across groups. For comparison of treatment efficacy according to *Leishmania* species, the main limitation is the small number of studies gathered. All freely available databases were evaluated covering a period of 30 years (1986–2016). In addition, studies with different designs were included, which made this review comprehensive and useful in revealing the lack of evidence required to support a therapeutic recommendation. On the other hand, there was no evidence to suggest publication bias, as estimated by the funnel plots. These results should not be understood as evidence to support TL management recommendations but as relevant information to guide future clinical studies. Trials designed in accordance with the current criteria and outcome standardization for CL [20] evaluating higher doses of the different azoles in different patient subgroups in different regions of the world, including in combination, may in the future base recommendations for its incorporation into the limited arsenal of alternatives for the treatment of leishmaniasis. At present, the quality of the available data does not allow to conclude on the benefit of the azole therapy for the treatment of the tegumentary form of leishmaniasis.

## Supporting information

S1 File Search strategy. (TIF)

S1 Table Adverse events enrolled in the azole arm in the Old World leishmaniasis studies. (DOCX)

S2 Table Adverse events enrolled in the azole arm in the New World leishmaniasis studies. (DOCX)

S3 Table Quality assessment of randomized controlled trials. (DOCX)

S4 Table The Newcastle-Ottawa Scale (NOS) for assessing the quality of nonrandomized studies. (DOCX)

S5 Table Summary assessments of the risk of bias for each important outcome (across domains) within and across studies. (DOCX)

S6 Table PRISMA checklist. (DOC)

S1 Fig Funnel plots for the compilation of (A) Initial response and (B) Final efficacy rate of all azoles.
(TIF)

S2 Fig Funnel plots for the compilation of Initial response and Final efficacy rate of each azole: (A) Ketoconazole, (B) Fluconazole and (C) Itraconazole.
(TIF)

S3 Fig Funnel plots for the compilation of Final efficacy rate of all azoles according (A) Old World and New World (B).
(TIF)

S4 Fig Dosage influence on effect of azoles on final cure rate of LT. (TIF)

S5 Fig Dosage influence on effect of each azole: (A) fluconazole, (B) ketoconazole and (C) itraconazole, on final efficacy rate of LT.
(TIF)

## References

[1] Alvar J, Vélez1 ID, Bern C, Herrero M, Desjeux P, Cano J, et al The WHO Leishmaniasis Control Team. Leishmaniasis Worldwide and Global Estimates of Its Incidence. PLoS One. 2012; 7(5), e35671 Epub 2012/05/31. doi: 10.1371/journal.pone.0035671 22693548 10.1371/journal.pone.0035671 PMC3365071

[2] Gurel MS, Yanik M, Simsek Z, Kati M, Karaman A. Quality of life instrument for Turkish people with skin diseases. Int J Dermatol. 2015; 44(11):933–8 doi: 10.1111/j.1365-4632.2004.02225.x 16336527 10.1111/j.1365-4632.2004.02225.x

[3] Kassi M, Kassi M, Afghan AK, Rehman R, Kasi PM. Marring Leishmaniasis: The Stigmatization and the Impact of Cutaneous Leishmaniasis in Pakistan and Afghanistan. PLoS Negl Trop Dis. 2008; 2(10):e259 Epub 2008/10/29. doi: 10.1371/journal.pntd.0000259 18958168 10.1371/journal.pntd.0000259 PMC2569210

[4] Chahed MK, Bellali H, Jemaa SB, Belaj T. Pscychological and Psicosocial consequences of zoonotic Cutaneous Leishmaniasis among Women in Tunisia: Preliminary Findings from an exploratory Study. PLoS Negl Trop Dis. 2016; 10 27;10(10):e0005090 eCollection 2016. doi: 10.1371/journal.pntd.0005090 27788184 10.1371/journal.pntd.0005090 PMC5082956

[5] Khatami A, Firooz A, Gorouhi F, Dowlati Y. Treatment of acute Old World cutaneous leishmaniasis: a systematic review of the randomized controlled trials. J Am Acad Dermatol. 2007; 57(2):335.e1–29. Epub 2007/03/06. doi: 10.1016/j.jaad.2007.01.016 17337090 10.1016/j.jaad.2007.01.016

[6] Tuon FF, Amato VS, Graf ME, Siqueira AM, Nicodemo AC, Amato Neto V. Treatment of New World cutaneous leishmaniasis—a systematic review with a meta-analysis. Int J Dermatol. 2008; 47(2):109–124. doi: 10.1111/j.1365-4632.2008.03417.x 18211479 10.1111/j.1365-4632.2008.03417.x

[7] Gonzalez U, Pinart M, Reveiz L, Alvar J. Interventions for Old World cutaneous leishmaniasis. Cochrane Database Syst Rev. 2008; 8(4):CD005067 doi: 10.1002/14651858.CD005067.pub3 18843677 10.1002/14651858.CD005067.pub3

[8] González U, Pinart M, Rengifo-Pardo M, Macaya A, Alvar J, Tweed JA. Interventions for American cutaneous and mucocutaneous leishmaniasis. Cochrane Database Syst Rev. 2009; 15(2): CD004834 doi: 10.1002/14651858.CD004834.pub2 19370612 10.1002/14651858.CD004834.pub2

[9] Reveiz L, Maia-Elkhoury AN, Nicholls RS, Romero GA, Yadon ZE. Interventions for American cutaneous and mucocutaneous leishmaniasis: a systematic review update. PLoS One. 2013; 8(4): e61843 doi: 10.1371/journal.pone.0061843 23637917 10.1371/journal.pone.0061843 PMC3639260

[10] Oliveira LF, Schubach AO, Martins MM, Passos SL, Oliveira RV, Marzochi MC, et al Systematic review of the adverse effects of cutaneous leishmaniasis treatment in the New World. Acta Trop. 2011 5; 118(2):87–96. Epub 2011/03/21. doi: 10.1016/j.actatropica.2011.02.007 21420925 10.1016/j.actatropica.2011.02.007

[11] WHO. Report of a meeting of the WHO Expert Committee on the Control of Leishmaniases, Geneva, Switzerland, 22–26 March 2010. WHO technical report series. 2010;(949).

[12] Beach DH, Goad LJ, Holz GG Jr. Effects of antimycotic azoles on growth and sterol biosynthesis of Leishmania promastigotes. Mol Biochem Parasitol. 1988; 31(2):149–62. 2847043 10.1016/0166-6851(88)90166-1

[13] Croft SL, Yardley V. Chemotherapy of leishmaniasis. Curr Pharmaceut Design 2002; 8(4): 319–342. 11860369 10.2174/1381612023396258

[14] Shokri A, Emami S, Fakhar M, Teshnizi SH, Keighobadi M. In vitro antileishmanial activity of novel azoles (3-imidazolylflavanones) against promastigote and amastigote stages of Leishmania major. Acta Trop. 2017; 167:73–78. Epub 2016 Dec 23. doi: 10.1016/j.actatropica.2016.12.027 28017860 10.1016/j.actatropica.2016.12.027

[15] Dogra J, Aneja N, Lal BB, Mishra SN.Cutaneous leishmaniasis in India. Clinical experience with itraconazole. Int J Dermatol. 1990; 29(9):661–2. 2177041 10.1111/j.1365-4362.1990.tb02593.x

[16] Saenz RE, Paz H, Berman JD. Efficacy of ketoconazole against Leishmania braziliensis panamensis cutaneous leishmaniasis. Am J Med. 1990; 89(2):147–55. 2166429 10.1016/0002-9343(90)90292-l

[17] Alrajhi AA, Ibrahim EA, De Vol EB, Khairat M, Faris RM, Maguire JH. Fluconazole for the treatment of cutaneous leishmaniasis caused by Leishmania major. N Engl J Med. 2002; 21;346(12):891–5. doi: 10.1056/NEJMoa011882 11907288 10.1056/NEJMoa011882

[18] Higgins JPT GSe. Cochrane Handbook for Systematic Reviews of Interventions Version 5.1.0 [updated March 2011]. The Cochrane Collaboration

[19] Moher D, Liberati A, Tetzlaff J, Altman DG. Preferred reporting items for systematic reviews and meta-analyses: the PRISMA statement. BMJ. 2009;339:b2535 doi: 10.1136/bmj.b2535 19622551 10.1136/bmj.b2535 PMC2714657

[20] Olliaro P, Vaillant M, Arana B, Grogl M, Modabber F, Magill A, et al Methodology of clinical trials aimed at assessing interventions for cutaneous leishmaniasis. PLoS Negl Trop Dis. 2013; 7(3):e2130 Epub 2013/04/05. doi: 10.1371/journal.pntd.0002130 23556016 10.1371/journal.pntd.0002130 PMC3605149

[21] Wells G, Shea B, O’connell D, Peterson J, Welch V, Losos M, et al. The Newcastle-Ottawa Scale (NOS) for assessing the quality of nonrandomised studies in meta-analyses. 2011. Available from: http://www.ohrica/programs/clinical_epidemiology/oxford asp. 2014.

[22] Begg CB, Mazumar M. Operating characteristics of a rank correlation test for publication bias. Biometrics. 1994; 50(4): 1088–1101. 7786990

[23] Restrepo M, Gomez ME. Tratamiento de la leishmaniasis cutanea con ketoconazol. Acta Med Colomb. 1987; 12(4): 294:7.

[24] Scorza JV, Hernández A, Villegas E, Márquez JC, Marcucci M. Efectividad del NysoralR (Ketoconazol) para el tratamiento de las leishmaniasis cutánea y cutáneomucosa en Trujillo, Venezuela. Bol. Dir. Malariol. Saneam. Amb. 1988; 28: 32–9.

[25] Santos MF, Ferreira SMB, Menezes JA.Tratamento da leishmaniose tegumentar americana pelo ketoconazol—Análise de 21 casos e estudo comparativo pareado com o antimoniato de N-metilglucamina (Glucantime). An Bras Dermatol. 1988; 63(6).

[26] al-Fouzan AS, al Saleh QA, Najem NM, Rostom MAI. Cutaneous leishmaniasis in Kuwait. Clinical experience with itraconazole. Int J Dermatol. 1991 7;30(7):519–21. 1663089 10.1111/j.1365-4362.1991.tb04878.x

[27] Norton SA, Frankenburg S, Klaus SN. Cutaneous leishmaniasis acquired during military service in the Middle East. Arch Dermatol. 1992 1;128(1):83–7. 1739291

[28] Navin TR, Arana BA, Arana FE, Berman JD, Chajón JF. Placebo-controlled clinical trial of sodium stibogluconate (Pentostam) versus ketoconazole for treating cutaneous leishmaniasis in Guatemala. J Infect Dis. 1992 3;165(3):528–34. 1311351 10.1093/infdis/165.3.528

[29] Singh KK, Kumar S, Singh VB, Mohan L, Mukhij. Role of ketoconazole in oriental sore. Indian J Dermatol Venereol Leprol. 1993; 59(3): 120–21

[30] Dogra J Aneja N. Leishmaniasis and itraconazole: a controlled clinical trial on cutaneous subtypes. Int J Antimicrob Agents. 1994; 4(4):309–11. 18611622 10.1016/0924-8579(94)90031-0

[31] Van den Enden E, Van Gompel A, Stevens A, Vandeghinste N, Le Ray D, Gigase P, De Beule K, Van den Ende J. Treatment of cutaneous leishmaniasis with oral itraconazole. Int J Dermatol. 1994;33(4):285–6. 8021092 10.1111/j.1365-4362.1994.tb01049.x

[32] Santos I, Santos IB, Montenegro D, Lemos ER, Pereira TS. The use of itraconazole in the treatment of 26 patients suffering from american cutaneous leishmaniasis. An Bras Dermatol. 1995; 70(2).

[33] Alsaleh QA, Dvorak R, Nanda A. Ketoconazole in the treatment of cutaneous leishmaniasis in Kuwait. Int J Dermatol. 1995; 34(7):495–7. 7591417 10.1111/j.1365-4362.1995.tb00622.x

[34] Singh S, Singh R, Sundar S. Failure of ketoconazole treatment in cutaneous leishmaniasis. Int J Dermatol. 1995; 34(2):120–1. 7737771 10.1111/j.1365-4362.1995.tb03595.x

[35] Dogra J, Saxena VN. Itraconazole and leishmaniasis: a randomised double-blind trial in cutaneous disease. Int J Parasitol. 1996;26(12):1413–5. 9024895 10.1016/s0020-7519(96)00128-2

[36] Momeni AZ, Jalayer T, Emamjomeh M, Bashardost N, Ghassemi RL, Meghdadi M, et al Treatment of cutaneous leishmaniasis with itraconazole. Randomized double-blind study. Arch Dermatol. 1996 7;132(7):784–6. 8678570

[37] Ozgoztasi O, Baydar I. A randomized clinical trial of topical paromomycin versus oral ketoconazole for treating cutaneous leishmaniasis in Turkey. Int J Dermatol. 1997; 36(1):61–3. 9071622 10.1046/j.1365-4362.1997.00022.x

[38] Viriyavejakul P, Viravan C, Riganti M, Punpoowong B. Imported cutaneous leishmaniasis in Thailand. Southeast Asian J Trop Med Public Health. 1997; 28(3):558–62. 9561608

[39] Ma Siddiqui, Ai-Mofadhi AM, Ai-Reshaid A, Ai-Rakban A, Ai-Jarba A, Kahtani H, et al Treatment of cutaneous leishmaniasis with itraconazole. J Dermatolog Treat. 1998; 9(4): 235–238. doi: 10.3109/09546639809160701

[40] Amato VS, Padilha ARS, Nicodemo AC, Duarte MIS, Valentini M, Uip DE, et al Use of itraconazole in the treatment of mucocutaneous leishmaniasis: A pilot study. Int J Infect Dis. 2000; 4(3):153–7. 11179919 10.1016/s1201-9712(00)90077-8

[41] Salmanpour R, Handjani F, Nouhpisheh MK. Comparative study of the efficacy of oral ketoconazole with intra-lesional meglumine antimoniate (Glucantime) for the treatment of cutaneous leishmaniasis. J Dermatolog Treat 12 (3), 159–162. doi: 10.1080/09546630152607899 12243707 10.1080/09546630152607899

[42] Calvopina M, Guevara AG, Armijos RX, Hashiguchi Y, Davidson RN, Cooper PJ. Itraconazole in the treatment of New World mucocutaneous leishmaniasis. Int J Dermatol. 2004; 43(9):659–63. doi: 10.1111/j.1365-4632.2004.02183.x. 15357745 10.1111/j.1365-4632.2004.02183.x

[43] Nassiri-Kashani M, Firooz A, Khamesipour A, Mojtahed F, Nilforoushzadeh M, Hejazi H, et al A randomized, double-blind, placebo-controlled clinical trial of itraconazole in the treatment of cutaneous leishmaniasis. J Eur Acad Dermatol Venereol. 2005;19(1):80–3. doi: 10.1111/j.1468-3083.2004.01133.x 15649196 10.1111/j.1468-3083.2004.01133.x

[44] Willard RJ, Jeffcoat AM, Benson PM, Walsh DS. Cutaneous leishmaniasis in soldiers from Fort Campbell, Kentucky returning from Operation Iraqi Freedom highlights diagnostic and therapeutic options. J Am Acad dermatol. 2005;52(6):977–87. doi: 10.1016/j.jaad.2005.01.109 15928615 10.1016/j.jaad.2005.01.109

[45] Morizot G, Delgiudice P, Caumes E, Laffitte E, Marty P, Dupuy A, et al Healing of Old World cutaneous leishmaniasis in travelers treated with fluconazole: drug effect or spontaneous evolution? Am J Trop Med Hyg. 2007;76(1):48–52. 17255228

[46] Rafaa M, Ingen-Housz-Oro S, Méry L, Le Turdu F, Wendling J, Pauwels C, Sigal-Grinberg M. Fluconazole in the treatment of cutaneous leishmaniasis in children. Ann Dermatol Venereol. 2007;134(8–9):682–3. 17925695 10.1016/s0151-9638(07)91833-5

[47] Saleem K, Rahman A. Comparison of oral itraconazole and intramuscular meglumine antimoniate in the treatment of cutaneous leishmaniasis. J Coll Physicians Surg Pak. 2007;17(12):713–6. 12.2007/JCPSP.713716. doi: 10.2007/JCPSP.713716 18182133

[48] Al-Mutairi N, Alshiltawy M, El Khalawany M, Joshi A, Eassa BI, Manchanda Y, et al Tropical medicine rounds: Treatment of Old World cutaneous leishmaniasis with dapsone, itraconazole, cryotherapy, and imiquimod, alone and in combination. Int J Dermatol. 2009;48(8):862–9. 19673049 10.1111/j.1365-4632.2008.04010.x

[49] Amato VS, Tuon FF, Imamura R, Abegão de Camargo R, Duarte MI, Neto VA. Mucosal leishmaniasis: description of case management approaches and analysis of risk factors for treatment failure in a cohort of 140 patients in Brazil. J Eur Acad Dermatol Venereol. 2009; 23(9):1026–34. Epub 2009/05/04. doi: 10.1111/j.1468-3083.2009.03238.x 19453817 10.1111/j.1468-3083.2009.03238.x

[50] Emad M, Hayati F, Fallahzadeh MK, Namazi MR. Superior efficacy of oral fluconazole 400 mg daily versus oral fluconazole 200 mg daily in the treatment of cutaneous leishmania major infection: a randomized clinical trial. J Am Acad Dermatol. 2011;64(3):606–8. doi: 10.1016/j.jaad.2010.04.014 21315963 10.1016/j.jaad.2010.04.014

[51] Sousa AQ, Frutuoso MS, Moraes EA, Pearson RD, Pompeu MM. High-dose oral fluconazole therapy effective for cutaneous leishmaniasis due to Leishmania (Vianna) braziliensis. Clin Infect Dis. 2011;53(7):693–5. doi: 10.1093/cid/cir496 21890773 10.1093/cid/cir496

[52] Silva CGL. Evaluation of therapeutic efficacy of fluconazole in human cutaneous leishmaniasis. Ph.D.Thesis, Universidade Federal do Ceará. 2012. Available from: http://www.repositorio.ufc.br/bitstream/riufc/5552/1/2012_tese_cglsilva.pdf

[53] Khan W, Zakai HA. Epidemiology, pathology and treatment of cutaneous leishmaniasis in taif region of saudi Arabia. Iran J Parasitol. 2014;9(3):365–73. 25678921 PMC4316568

[54] Prates FV, Dourado ME, Silva SC, Schriefer A, Guimarães LH, Brito MD, et al Fluconazole in the Treatment of Cutaneous Leishmaniasis Caused by Leishmania braziliensis: A Randomized Controlled Trial. Clin Infect Dis. 2017 1 1;64(1):67–71. Epub 2016/11/01. doi: 10.1093/cid/ciw662 27803094 10.1093/cid/ciw662

[55] Dedet JP, Jamet P, Esterre P, Ghipponi PM, Genin C, Lalande G. Failure to cure Leishmania braziliensis guyanensis cutaneous leishmaniasis with oral ketoconazole. Trans R Soc Trop Med Hyg. 1986;80(1):176 3726992 10.1016/0035-9203(86)90239-7

[56] Zahaf A, Sevestre H, Fraitag S, Shoutet P, Smadja A. Efficacite du ketoconazole dans le traitement de la leishmaniose cutanee a leishmania major en Tunisia. Med Maladies Infect. 1987; 17(6–7):420–421.

[57] Weinrauch L, Livshin R, Even-Paz Z, El-On J. Efficacy of ketoconazole in cutaneous leishmaniasis. Arch Dermatol Res. 1983;275(5):353–4. 6318670 10.1007/BF00417211

[58] Jolliffe DS. Cutaneous leishmaniasis from Belize—treatment with ketoconazole. Clin Exp Dermatol. 1986 1;11(1):62–8. 3708895 10.1111/j.1365-2230.1986.tb00425.x

[59] Netto EM, Costa JML; Vieira JB, Marsden PD. An attempt to treat skin ulcer caused by Leishmania braziliensis braziliensis with ketoconazole. Rev Soc Bras Med Trop. 1989; 22(2): 105–6. 2638483

[60] Pita JC, Teitelman ML, Gallardo JLB, Ocampo WC, Mestanza KMP, Tirado EQ. Tratamiento de leishmaniasis cutánea andina con ketoconazol en dos zonas de alta incidência del Departamento de Amazonas: Reporte de Casos. Rev. Méd. Hered. 2002; 13(4): 144–147

[61] Baron S, Laube S, Raafat F, Moss C. Cutaneous leishmaniasis in a Kosovan child treated with oral fluconazole. Clin Exp Dermatol. 2004;29(5):546–7. doi: 10.1111/j.1365-2230.2004.01561.x 15347346 10.1111/j.1365-2230.2004.01561.x

[62] Consigli J, Danielo C, Gallerano V, Papa M, Guidi A. Cutaneous leishmaniasis: successful treatment with itraconazole. International Journal of Dermatology 2006, 45, 46–49. doi: 10.1111/j.1365-4632.2004.02429.x 16426375 10.1111/j.1365-4632.2004.02429.x

[63] Larosa E, Cauwenbergh G, Cilli P et al Itraconazole pharmacokinetics in female genital tract: plasma and tissue levels in patients undergoing hysterectomy after a single dose of 200 mg itraconazole. Eur J Obstet Gynecol Reprod Biol. 1986; 23:85–89. 3023155 10.1016/0028-2243(86)90109-7

[64] Heykants J, Van Peer A, Van de Velde V, Van Rooy P, Meuldermans W, Lavrijsen K, et al The clinical pharmacokinetics of itraconazole: an overview. Mycoses. 1989;32 Suppl 1:67–87. 2561187 10.1111/j.1439-0507.1989.tb02296.x

[65] Vita GF, Pereira MA, Ferreira I, Sanavria A, Barbosa CG, Aurnheimer RC, et al Status of the american tegumentary leishmaniasis in the state of Rio de Janeiro, Brazil, from 2004 to 2013. Rev Inst Med Trop Sao Paulo. 2016; 22;58:71 doi: 10.1590/S1678-9946201658071 27680176 10.1590/S1678-9946201658071 PMC5048642

[66] Mosimann V, Neumayr A, Hatz C, Blum JA.Cutaneous leishmaniasis in Switzerland: first experience with species-specific treatment. Infection. 2013;41(6):1177–82. Epub 2013/08/09. doi: 10.1007/s15010-013-0500-5 23835701 10.1007/s15010-013-0500-5

[67] Zeegelaar JE, Steketee WH, van Thiel PP, Wetsteyn JC, Kager PA, Faber WR. Changing pattern of imported cutaneous leishmaniasis in the Netherlands. Clin Exp Dermatol. 2005;30(1):1–5. doi: 10.1111/j.1365-2230.2004.01677.x 15663490 10.1111/j.1365-2230.2004.01677.x

[68] Cota GF, de Sousa MR, Fereguetti TO, Saleme PS, Alvarisa TK, Rabello A. The Cure Rate after Placebo or No Therapy in American Cutaneous Leishmaniasis: A Systematic Review and Meta-Analysis. PLoS One. 2016;11(2):e0149697 Epub 2016/02/20. doi: 10.1371/journal.pone.0149697 26894430 10.1371/journal.pone.0149697 PMC4760744

[69] Moher D, Schulz KF, Altman DG, for the CONSORT Group. The CONSORT Statement: revised recommendations for improving the quality of reports of parallel-group randomized trials. The Lancet. 2001; 357(9263): 1191–94. . 11323066

