# Supplementary figures and images for: Efficacy of azole therapy for tegumentary leishmaniasis: A systematic review and meta-analysis
Source: PLoS One. 2017 Oct 9;12(10):e0186117. doi: 10.1371/journal.pone.0186117 (PMC5633178; doi:10.1371/journal.pone.0186117)

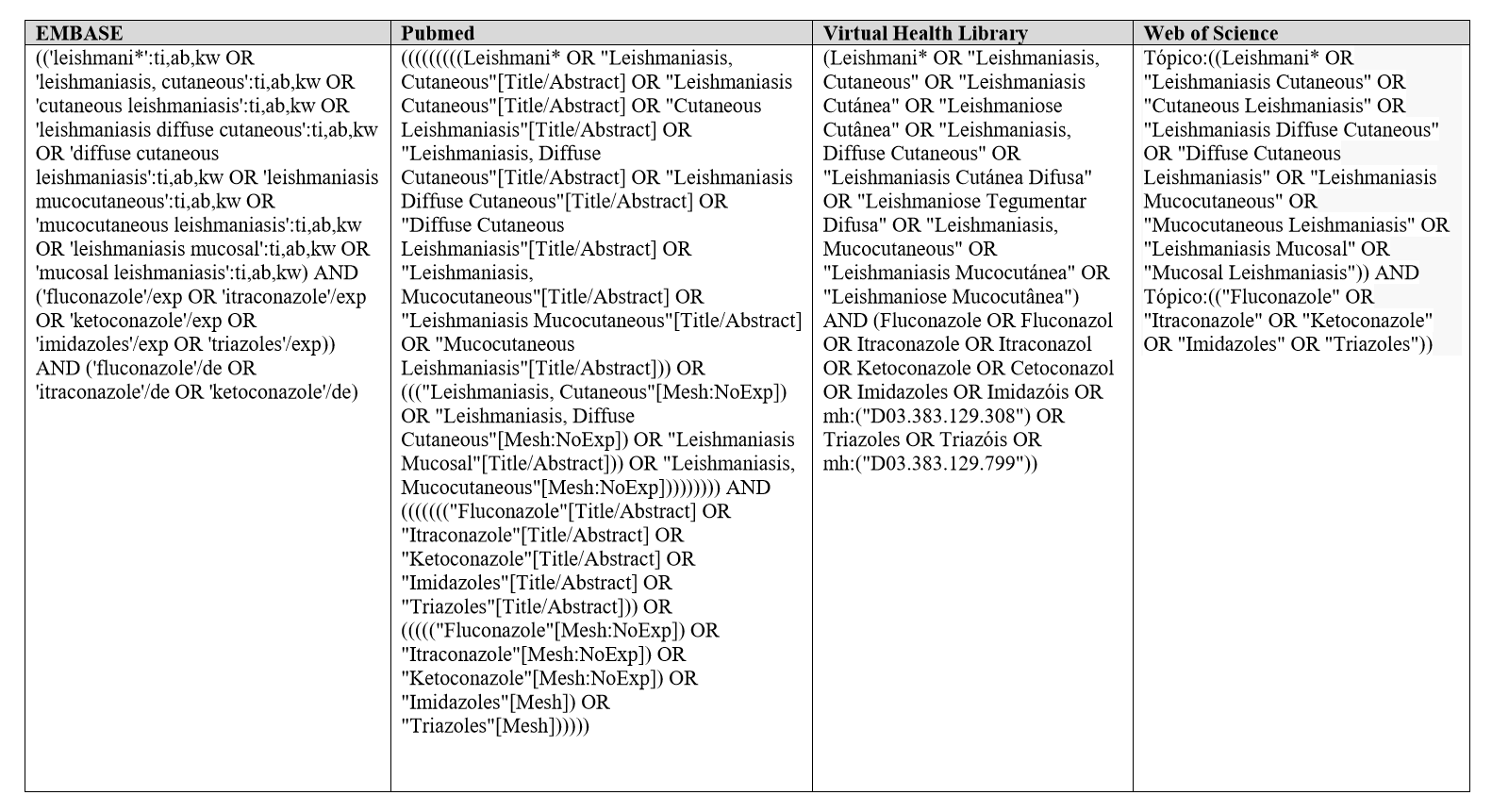

Supplement: S1 File — (TIF) [file pone.0186117.s001.tif]

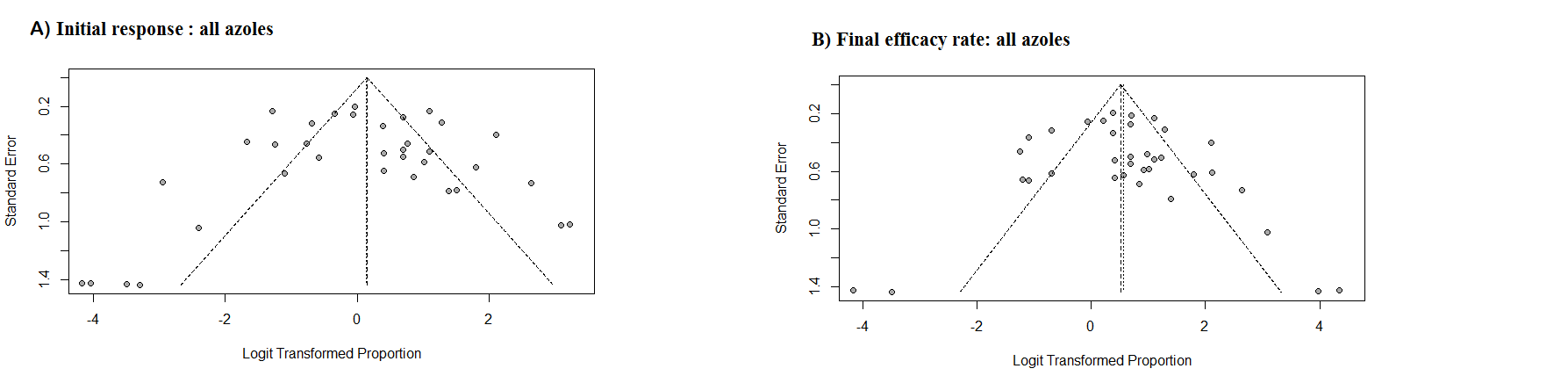

Supplement: S1 Fig — Funnel plots for the compilation of (A) Initial response and (B) Final efficacy rate of all azoles. (TIF) [file pone.0186117.s008.tif]

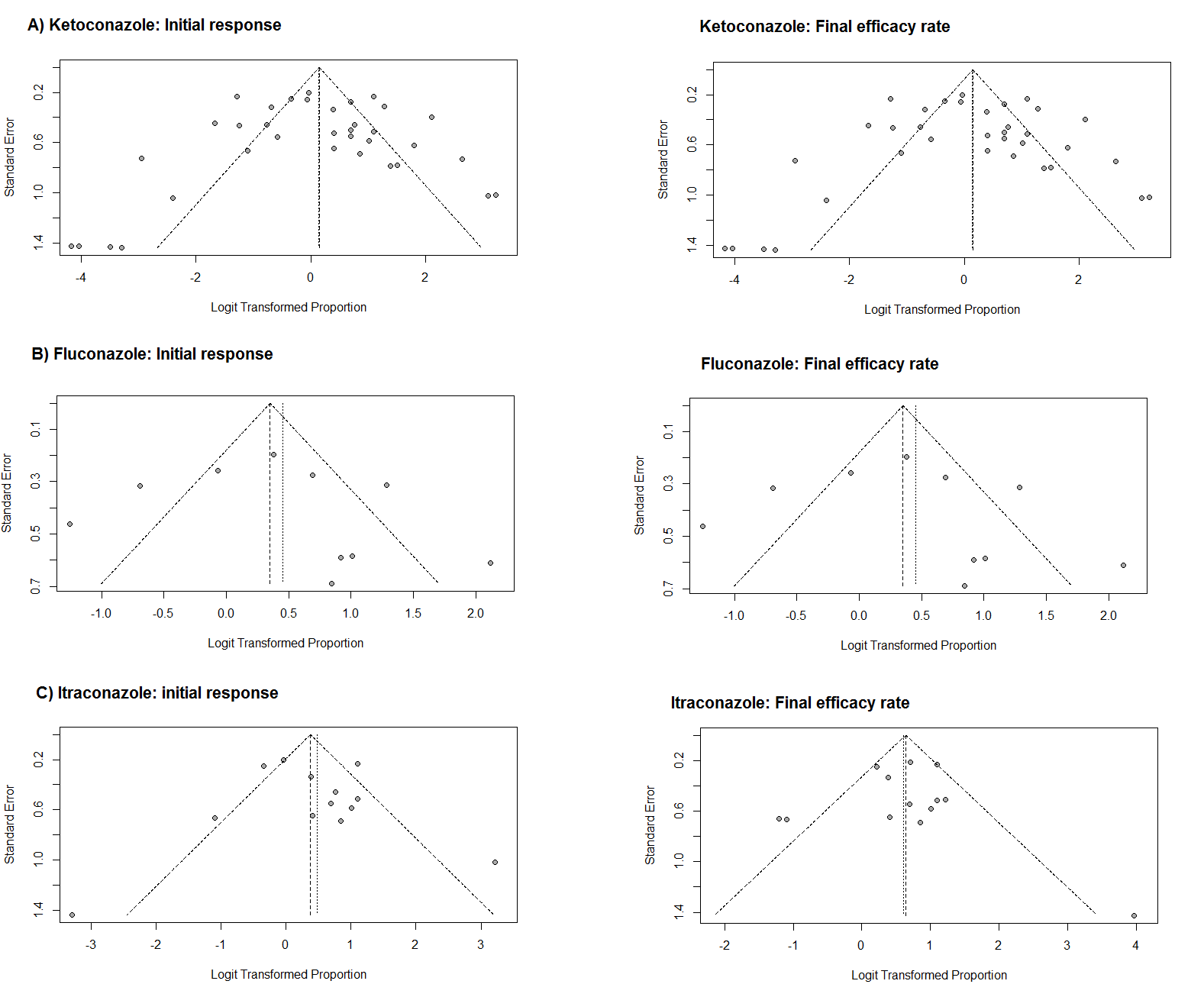

Supplement: S2 Fig — Funnel plots for the compilation of Initial response and Final efficacy rate of each azole: (A) Ketoconazole, (B) Fluconazole and (C) Itraconazole. (TIF) [file pone.0186117.s009.tif]

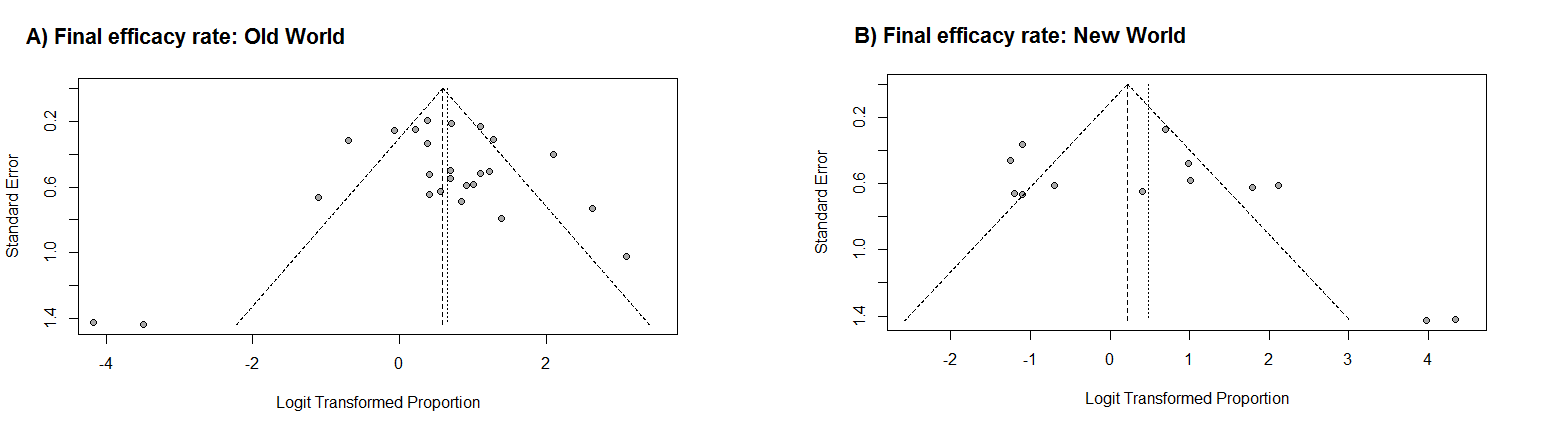

Supplement: S3 Fig — Funnel plots for the compilation of Final efficacy rate of all azoles according (A) Old World and New World (B). (TIF) [file pone.0186117.s010.tif]

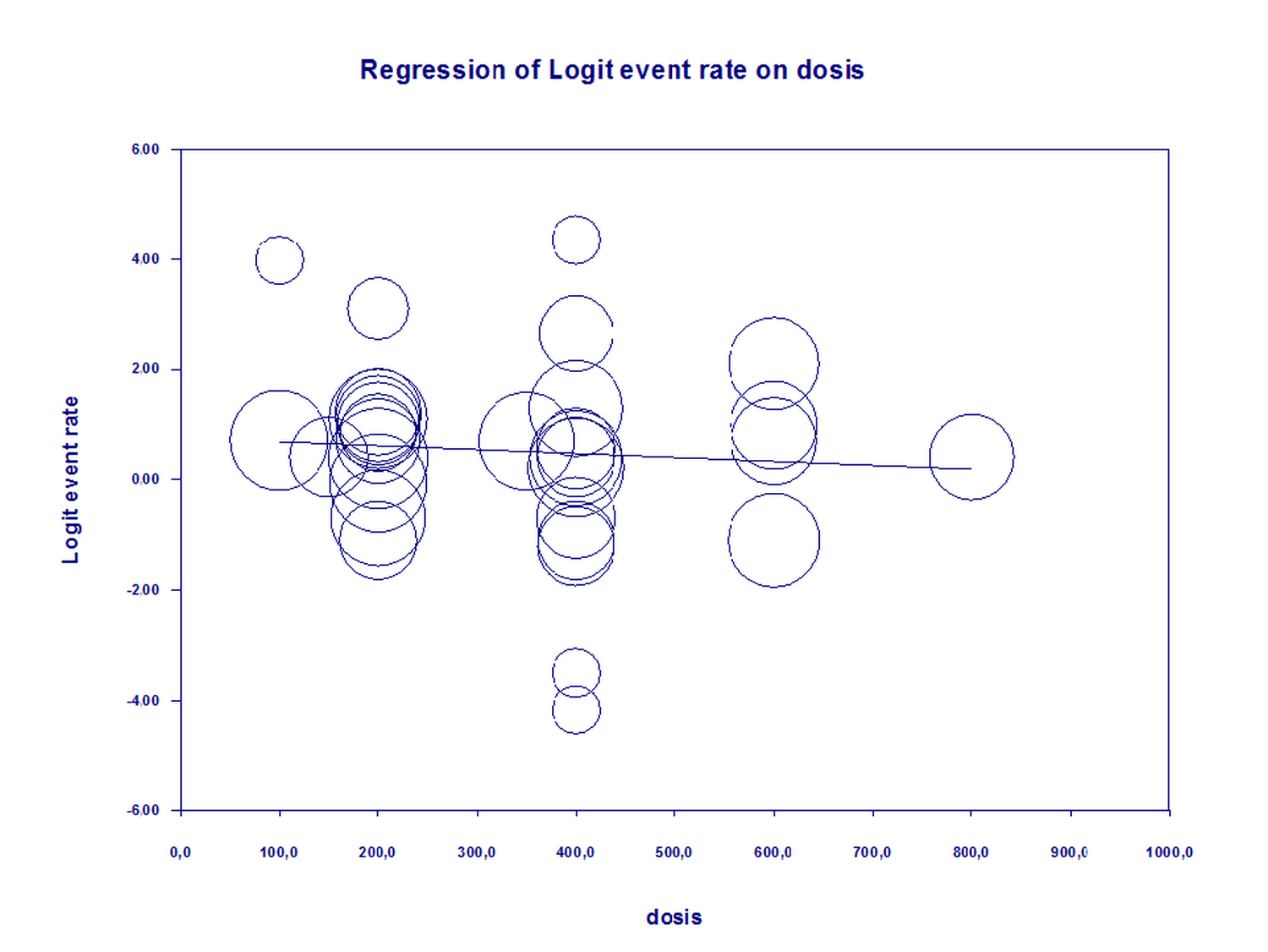

Supplement: S4 Fig — (TIF) [file pone.0186117.s011.tif]

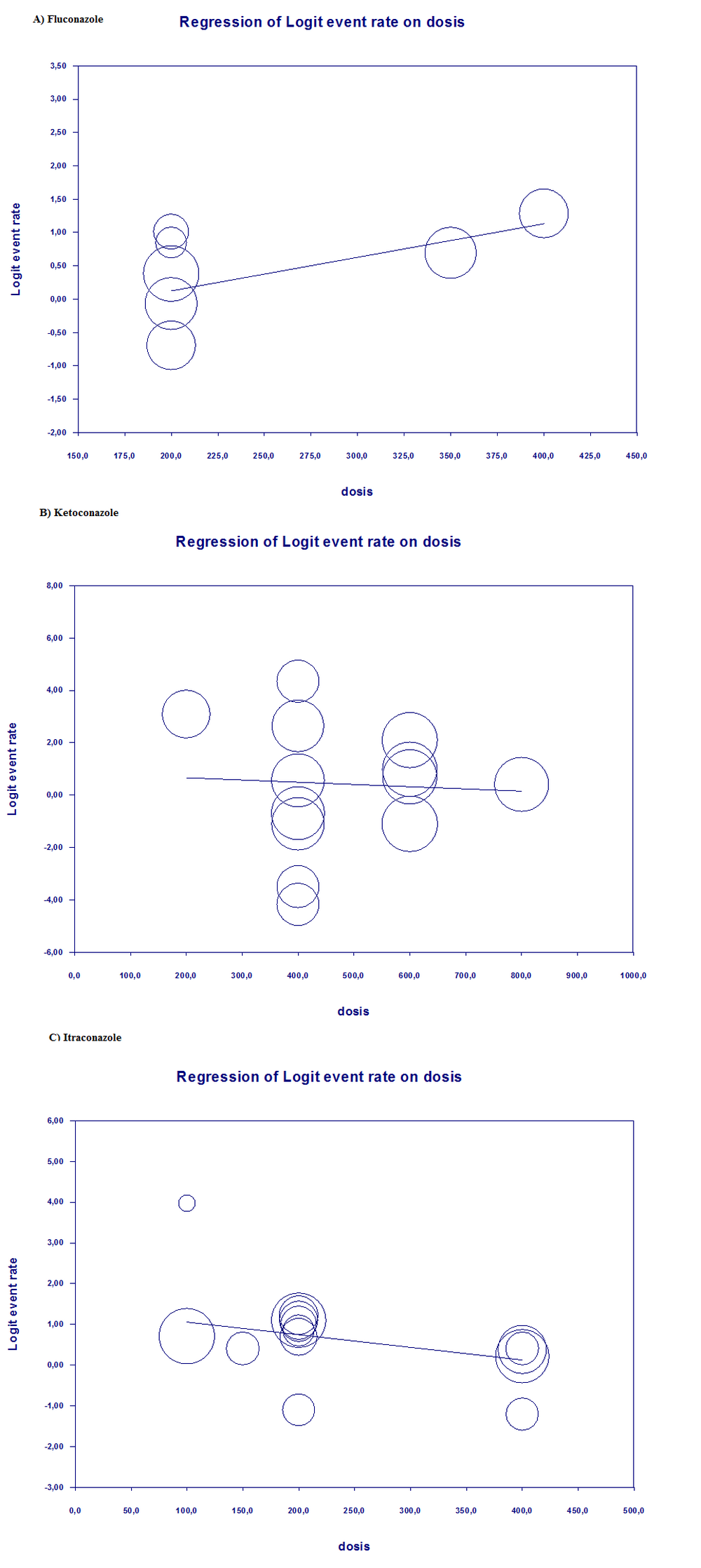

Supplement: S5 Fig — Dosage influence on effect of each azole: (A) fluconazole, (B) ketoconazole and (C) itraconazole, on final efficacy rate of LT. (TIF) [file pone.0186117.s012.tif]
