# Supplementary material for: Efficacy of azole therapy for tegumentary leishmaniasis: A systematic review and meta-analysis
Source: PLoS One. 2017 Oct 9;12(10):e0186117. doi: 10.1371/journal.pone.0186117 (PMC5633178; doi:10.1371/journal.pone.0186117)
Supplement: S1 Table — (DOCX) [file pone.0186117.s002.docx]

**S1 Table. Adverse events enrolled in the azole arm in the Old World leishmaniasis studies**

| **Year, Author** | **Azole drug used**  **(number of patients treated)** | **Number of patients presenting adverse events reported** | **Number of patients presenting liver exams abnormalities** | **Number of patients presenting dizziness** | **Number of patients presenting malaise** | **Number of patients presenting nausea** | **Number of patients presenting vomiting** | **Number of patients presenting headache** | **Number of patients presenting myalgia** | **Number of patients presenting abdominal pain** | **Other events (n)** |
| --- | --- | --- | --- | --- | --- | --- | --- | --- | --- | --- | --- |
| **1987, Zahaf** | KTZ (10) | 1 | 1 | 0 | 0 | 0 | 0 | 0 | 0 | 0 | 0 |
| **1990, Dogra** | ITCZ (15) | 3 | 0 | 3 | 0 | 0 | 0 | 3 | 0 | 0 | 0 |
| **1991, Al-Fouzan** | ITCZ (15) | 3 | 1 | 0 | 0 | 2 | 0 | 2 | 0 | 0 | 0 |
| **1992, Norton** | KTZ (23) | NR | NR | NR | NR | NR | NR | NR | NR | NR | NR |
| **1993, Singh** | KTZ (30) | 1 | 0 | 0 | 0 | 0 | 0 | 0 | 0 | 0 | Jaundice (1) |
| **1994, Dogra** | ITCZ (20) | 3 | 3 | 0 | 0 | 0 | 0 | 0 | 0 | 0 | 0 |
| **1994, Enden** | ITCZ (22) | 5 | 1 | 0 | 0 | 0 | 2 | 0 | 0 | 2 | Temporary hair loss (2) |
| **1995, Alsaleh** | KTZ (18)^a^ | 1 | 0 | 0 | 0 | 0 | 0 | 0 | 0 | 0 | Serum triglycerides elevation (1) |
| **1995, Alsaleh** | KTZ (15)^b^ | 1 | 0 | 0 | 0 | 1 | 1 | 0 | 0 | 0 | 0 |
| **1995, Singh** | KTZ (16) | NR | NR | NR | NR | NR | NR | NR | NR | NR | NR |
| **1996, Dogra** | ITCZ (10) | 2 | 1 | 0 | 0 | 1 | 0 | 0 | 0 | 0 | 0 |
| **1996, Momemi** | ITCZ (65) | 6 | 0 | 0 | 0 | 6 | 0 | 0 | 0 | 6 | 0 |
| **1997, Ozgoztasi** | KTZ (32) | 0 | 0 | 0 | 0 | 0 | 0 | 0 | 0 | 0 | 0 |
| **1997, Viriyavejakul** | KTZ (11) | 0 | 0 | 0 | 0 | 0 | 0 | 0 | 0 | 0 | 0 |
| **1998, Siddiqui** | ITCZ (37) | 0 | 0 | 0 | 0 | 0 | 0 | 0 | 0 | 0 | 0 |
| **2001, Salmanpour** | KTZ (64) | NR | NR | NR | NR | NR | NR | NR | NR | NR | NR |
| **2002, Alrajhi** | FCZ (106) | NR | NR | NR | NR | NR | NR | NR | NR | NR | NR |
| **2005, Nassiri-Kashani** | ITCZ (100) | 5 | NR | NR | NR | NR | NR | NR | NR | NR | NR |
| **2005, Willard** | FCZ (15) | 1 | 1 | 0 | 0 | 0 | 0 | 0 | 0 | 0 | 0 |
| **2007, Morizot** | FCZ (45) | 4 | 1 | 0 | 0 | 3 | 0 | 0 | 0 | 2 | 0 |
| **2007, Rafaa** | FCZ (14) | 1 | 1 | 0 | 0 | 0 | 0 | 0 | 0 | 0 | 0 |
| **2007, Saleem** | ITCZ (100) | 12 | 12 | 0 | 0 | 0 | 0 | 0 | 0 | 0 | 0 |
| **2009, Al-Mutairi** | ITCZ (12) | NR | NR | NR | NR | NR | NR | NR | NR | NR | NR |
| **2011, Emad** | FCZ (60)^c^ | 0 | 0 | 0 | 0 | 0 | 0 | 0 | 0 | 0 | 0 |
| **2011, Emad** | FCZ (60)^d^ | NR | 1 | 0 | 0 | 10 | 0 | 0 | 0 | 0 | Serum creatinine elevation (1)  Cheilitis (45) |
| **2014, Khan** | FCZ (10) | NR | NR | 0 | 0 | 2 | 0 | 2 | 0 | 0 | Serum creatinine elevation (NR) |
| **2014, Khan** | ITCZ (10) | NR | NR | 0 | 0 | 1 | 0 | 0 | 0 | 0 | Serum creatinine elevation (NR) |

^a^: 600 mg/once; ^b^: 800 mg/once; ^c^: 100 mg/twice; ^d^: 200 mg/twice
