# Supplementary material for: Efficacy of azole therapy for tegumentary leishmaniasis: A systematic review and meta-analysis
Source: PLoS One. 2017 Oct 9;12(10):e0186117. doi: 10.1371/journal.pone.0186117 (PMC5633178; doi:10.1371/journal.pone.0186117)
Supplement: S2 Table — (DOCX) [file pone.0186117.s003.docx]

**S2 Table. Adverse events enrolled in the azole arm in the New World leishmaniasis studies**

| **Year, Author** | **Azole drug used**  **(number of patients treated)** | **Number of patients presenting adverse events reported** | **Number of patients presenting worsening liver function** | **Number of patients presenting dizziness** | **Number of patients presenting malaise** | **Number of patients presenting nausea** | **Number of patients presenting vomiting** | **Number of patients presenting headache** | **Number of patients presenting myalgia** | **Number of patients presenting abdominal pain** | **Other events (n)** |
| --- | --- | --- | --- | --- | --- | --- | --- | --- | --- | --- | --- |
| **1986, Dedet** | KTZ (12) | 9 | 0 | 0 | 0 | 0 | 0 | 0 | 0 | 2 | Astenia (6)  Sleepness (2)  Nervousness (2) |
| **1987, Restrepo** | KTZ (12) | 0 | 0 | 0 | 0 | 0 | 0 | 0 | 0 | 0 | 0 |
| **1988, Scorza** | KTZ (38) | NR | 6 | 0 | 0 | 0 | 0 | 0 | 0 | 0 | 0 |
| **1988, Santos** | KTZ (21) | 3 | 4 | 0 | 0 | 1 | 1 | 0 | 0 | 0 | 0 |
| **1990, Saenz** | KTZ (22) | 22 | 6 | 0 | 1 | 1 |  | 4 |  | 2 | Fever (2)  Decreased testosterone levels (22) |
| **1992, Navin** | KTZ (40) | 8 | 0 | 1 | 0 | 2 | 0 | 2 | 0 | 2 | Rash (1) |
| **1995, Santos** | ITCZ (26) | NR | 11 | 0 | 0 | 4 | 4 | 0 | 0 | 0 | Gastrointestinal disorders (4) |
| **2000, Amato** | ITCZ (10) | 0 | 0 | 0 | 0 | 0 | 0 | 0 | 0 | 0 | 0 |
| **2004, Calvopina** | ITCZ (13) | 0 | 0 | 0 | 0 | 0 | 0 | 0 | 0 | 0 | 0 |
| **2009, Amato** | ITCZ (10) | NR | NR | NR | NR | NR | NR | NR | NR | NR | NR |
| **2011, Sousa** | FCZ (28) | 1 | 0 | 0 | 0 | 1 | 0 | 0 | 0 | 0 | 0 |
| **2012, da Silva** | FCZ (60) | 0 | 0 | 0 | 0 | 0 | 0 | 0 | 0 | 0 | 0 |
| **2016, Prates** | FCZ (27) | 11 | 0 | 6 | 1 | 3 | 1 | 2 | 1 | 0 | 0 |
