# Supplementary material for: Efficacy of azole therapy for tegumentary leishmaniasis: A systematic review and meta-analysis
Source: PLoS One. 2017 Oct 9;12(10):e0186117. doi: 10.1371/journal.pone.0186117 (PMC5633178; doi:10.1371/journal.pone.0186117)
Supplement: S3 Table — (DOCX) [file pone.0186117.s004.docx]

**S3 Table. Quality assessment of randomized controlled trials**

| **Year, Author** | **Double- blind** | **Concealment of treatment allocation** | **Blinding of outcome assessment** | **Intention-to-treat analysis** |
| --- | --- | --- | --- | --- |
| **1990, Dogra** | No | No | No | Yes |
| **1990, Saenz** | No | Yes | No | Yes |
| **1991, Al-Fouzan** | No | No | No | Yes |
| **1992, Navin** | No | Yes | Yes | No |
| **1994, Dogra** | No | No | No | Yes |
| **1996, Dogra** | Yes | No | No reported | Yes |
| **1996, Momemi** | Yes | Yes | No reported | No |
| **1997, Ozgoztasi** | No | No | No | Yes |
| **2001, Salmanpour** | No | No | No | Yes |
| **2002, Alrajhi** | Yes | Yes | No reported | Yes |
| **2005, Nassari-Kashani** | Yes | Yes | No reported | Yes |
| **2011, Emad** | No | No | No | Yes |
| **2012, da Silva** | No | Yes | Yes | Yes |
| **2016, Prates** | No | Yes | Yes | Yes |
