# Supplementary material for: Efficacy of azole therapy for tegumentary leishmaniasis: A systematic review and meta-analysis
Source: PLoS One. 2017 Oct 9;12(10):e0186117. doi: 10.1371/journal.pone.0186117 (PMC5633178; doi:10.1371/journal.pone.0186117)
Supplement: S4 Table — (DOCX) [file pone.0186117.s005.docx]

**S4 Table. The Newcastle-Ottawa Scale (NOS) for assessing the quality of nonrandomized studies**

| **Non RTC studies**  **Year, author** | **Selection** | | | **Comparability** | | | **Assessment of Outcome** | | | **Total Quality score** |
| --- | --- | --- | --- | --- | --- | --- | --- | --- | --- | --- |
|  | Representativeness of the patients submitted to treatment of interest | Selection of the patients submitted to the control treatment | Ascertainment of the therapeutic option | Demonstration that outcome of interest was not present at start of study | Comparability between patients in different treatment arms – main factor: age | Comparability between patients in different treatment arms – secondary factor: number of lesions | Assessment of outcome with independency | Adequacy of the follow up length  (to assess outcome) ^a^ | Lost to follow up acceptable (less than 10% and reported) |  |
| **1986, Dedet** |  |  |  | * |  |  |  | * | * | 3 |
| **1987, Zahaf** |  |  |  | * |  |  |  |  | * | 2 |
| **1988, Santos** |  | * |  | * |  |  |  | * | * | 4 |
| **1987, Restrepo** |  | * |  | * |  |  |  |  |  | 2 |
| **1988, Scorza** |  |  |  | * |  |  |  | * | * | 3 |
| **1992, Norton** |  |  |  | * |  |  |  | * | * | 3 |
| **1993, Singh** |  |  |  | * |  |  |  | * | * | 3 |
| **1994, Enden** |  |  |  | * |  |  |  | * |  | 2 |
| **1995, Santos** |  |  |  | * |  |  |  | * | * | 3 |
| **1995, Singh** |  |  |  | * |  |  |  | * |  | 2 |
| **1995, Alsaleh** | * | * |  | * | * | * |  | * |  | 6 |
| **1997, Viriyavejakul** | * |  |  | * |  |  |  | * | * | 4 |
| **1998, Sidiqui** |  | * |  | * |  | * |  |  | * | 4 |
| **2000, Amato** | * |  |  | * |  |  |  | * | * | 4 |
| **2004, Calvopina** | * |  | * | * |  |  |  | * | * | 5 |
| **2005, Willard** |  |  |  | * |  |  |  |  | * | 2 |
| **2007, Saleem** | - | - | - | - |  |  | - | - | - | - |
| **2007, Rafaa** |  |  |  | * |  |  |  | * | * | 3 |
| **2007, Morizot** | * |  |  | * |  |  |  | * |  | 3 |
| **2009, Al-Mutairi** | * | * |  | * |  |  |  | * | * | 5 |
| **2009, Amato** | * |  |  | * |  |  |  | * | * | 4 |
| **2011, Souza** | * |  |  | * |  |  |  |  | * | 3 |
| **2014, Khan** | * | * |  | * |  |  |  | * | * | 5 |

^a^  Adequacy of the follow up length: cure assessment performed at least 74 days after initiation of therapy (1 star)
