# Supplementary material for: Efficacy of azole therapy for tegumentary leishmaniasis: A systematic review and meta-analysis
Source: PLoS One. 2017 Oct 9;12(10):e0186117. doi: 10.1371/journal.pone.0186117 (PMC5633178; doi:10.1371/journal.pone.0186117)
Supplement: S5 Table — (DOCX) [file pone.0186117.s006.docx]

**S5 Table.** Summary assessments of the risk of bias for each important outcome (across domains) within and across studies

| **Outcome** | **Relevant domains within a study group (number of studies)** | | | | | | | | | **Global risk of bias** |
| --- | --- | --- | --- | --- | --- | --- | --- | --- | --- | --- |
| Cure rate according to azole | Cure criteria adopted  Patient recruitment process  Outcome reporting | **ITCZ**  **(14)** | | **KTZ**  **(14)** | | | **FCZ**  **(9)** | | | High risk of bias mainly represented by differences in the cure criteria adopted across studies groups. |
|  |  | **8 +/ 14**  4 ++/ 14  2 +++/ 14  6 +/ 14  7 ++/ 14  1 +++/ 14  14 ++/ 14 | | **9 +/ 14**  4 ++/14  1 +++ /14  4 +/ 14  8 ++/ 14  2 +++/ 14  1 +/ 14  13 ++/ 14 | | | 4 ++/ 9  **5 +++/ 9**  4 +/ 9  4 ++/ 9  1 +++/ 9  2 +/ 9  7 ++/ 9 | | |  |
| Cure rate according to Leishmania species | Cure criteria adopted  Patient recruitment process  Outcome reporting | ***L. major* (1)** | ***L. braziliensis***  **(5)** | | ***L. tropica***  **(2)** | ***L. Mexicana***  **(1)** | | ***L. infantum***  **(1)** | ***L. donovani* (2)** | The main limitation is the small number of studies in each group. |
|  |  | 1 +/ 1  1 ++/ 1  1 ++/ 1 | 3 +/ 5  1 ++/ 5  1 +++/ 5  3 +/ 5  2 ++/ 5  3 +/ 5  2 ++/ 5 | | 2 +/ 2  2 ++/ 2  2 ++/ 2 | 1 +/ 1  1+/ 1  1 +/1 | | 1+/ 1  1 ++/1  1 ++/ 1 | 2 +/2  2 ++/2  2 ++/2 |  |
| Cure rate according to endemic region | Cure criteria  Adopted  Patient recruitment process  Outcome reporting | **Old World**  **(24)** | | | **New World**  **(13)** | | | | | Moderate risk of bias mainly represented by differences in the cure criteria adopted across studies groups. |
|  |  | **12 +/ 24**  11 ++/ 24  1 +++/ 24  10 +/ 24  10 ++/ 24  4 +++/ 24  24 ++/ 24 | | | **10 +/ 13**  1 ++/ 13  2 +++/ 13  4 +/ 13  8 ++/ 13  1 +++/ 13  3 +/ 13  10 ++/ 13 | | | | |  |

I**TCZ:** itraconazole; **FCZ:** fluconazole; **KTZ:** ketoconazole

Cure criteria adopted: parasitological (+); not reported (++); clinical (+++)

Patient recruitment process: randomization (+); prospective inclusion, aleatory (++); retrospective report (+++)

Outcome reporting: blinding assessment/no selective reporting (+); no blinding/no selective reporting (++); selective reporting (+++)
